# Robust Satisficing Decision Making for Unmanned Aerial Vehicle Complex Missions under Severe Uncertainty

**DOI:** 10.1371/journal.pone.0166448

**Published:** 2016-11-11

**Authors:** Xiaoting Ji, Yifeng Niu, Lincheng Shen

**Affiliations:** College of Mechatronics and Automation, National University of Defense Technology, Changsha, Hunan, China; Southwest University, CHINA

## Abstract

This paper presents a robust satisficing decision-making method for Unmanned Aerial Vehicles (UAVs) executing complex missions in an uncertain environment. Motivated by the info-gap decision theory, we formulate this problem as a novel robust satisficing optimization problem, of which the objective is to maximize the robustness while satisfying some desired mission requirements. Specifically, a new info-gap based Markov Decision Process (IMDP) is constructed to abstract the uncertain UAV system and specify the complex mission requirements with the Linear Temporal Logic (LTL). A robust satisficing policy is obtained to maximize the robustness to the uncertain IMDP while ensuring a desired probability of satisfying the LTL specifications. To this end, we propose a two-stage robust satisficing solution strategy which consists of the construction of a product IMDP and the generation of a robust satisficing policy. In the first stage, a product IMDP is constructed by combining the IMDP with an automaton representing the LTL specifications. In the second, an algorithm based on robust dynamic programming is proposed to generate a robust satisficing policy, while an associated robustness evaluation algorithm is presented to evaluate the robustness. Finally, through Monte Carlo simulation, the effectiveness of our algorithms is demonstrated on an UAV search mission under severe uncertainty so that the resulting policy can maximize the robustness while reaching the desired performance level. Furthermore, by comparing the proposed method with other robust decision-making methods, it can be concluded that our policy can tolerate higher uncertainty so that the desired performance level can be guaranteed, which indicates that the proposed method is much more effective in real applications.

## 1 Introduction

Over the past decades, Unmanned Aerial Vehicles (UAVs) have been extensively employed in many civil and military applications, such as search and rescue in the hazardous environment [1, 2], environment surveillance [3], 3D terrain reconstruction [4], climate research [5], and ground reconnaissance [6]. Various types of missions require high autonomy for UAVs to make proper decisions in complex environments. These complex missions may specify constraints on order, relative time, goal, safety, etc. Therefore, high-level mission specifications using temporal logics have been employed to improve the expressiveness of complex missions in recent years, such as the Linear Temporal Logic (LTL) [7–9]. The LTL provides a formal high-level framework to specify the complex mission with a natural encoding of Boolean and temporal operators, and atomic propositions, which will help enhance the interaction with UAVs and realize a better understanding of the behaviors of UAVs. For instance, a complex mission performed by an UAV can be described as “Take off from home A, search regions B, C, and D in a given order while always avoiding the unsafe regions F, and finally return to A”. Moreover, most missions for UAVs take place in complex and uncertain environments, which will result in actuation or sensing uncertainties for the UAVs. How to make proper decisions in face of uncertainties is an important aspect for UAVs decision making. Motivated by this situation, some researchers have studied the Markov Decision Process (MDP), which provides a general mathematical framework for sequential decision making under uncertainty [10]. MDPs use transition probabilities to model behavior uncertainties, which are caused by actuation errors or environmental disturbances. Considering the complex missions for the UAVs in a complex and uncertain environment, the synthesizing control method by combining the MDP with LTL have been studied. The decision making problem for the UAV executing a complex mission can be translated to generate a policy for the MDP in order to satisfy the LTL specifications. Many synthesizing control algorithms [11–13] based on the model checking theory [14, 15] have been provided in order to find an optimal control policy for the MDP with maximal probability of satisfying the LTL specifications.

However, when a real UAV system is abstracted to the MDP, it may lead to modeling uncertainties. And it is often prohibitively costly or even infeasible to obtain accurate transition probabilities in practice. Thus the estimated transition probabilities may deviate far from the true value due to errors in the abstraction process. Based on the existing works, it turns out that the optimal policy is often quite sensitive to even minor errors in the transition probabilities [16]. Therefore, the assumption that the transition probabilities are exact, when in fact they are uncertain, can lead to suboptimal decisions, or even degradation of system performance [17]. For synthesizing the MDP with LTL specifications, the uncertain transition probabilities will lead to a low probability of satisfying the LTL specifications, which means it will increase risks such as UAVs destruction and mission failure. So it is very important that the UAV must make robust decisions with respect to the uncertain transition probabilities of the MDP. Many researchers have devoted efforts to modeling the uncertain transition probability and mitigating its effect on the resulting policy in order to satisfy the mission requirements. The study of the MDP with uncertain transition probabilities, which is called an uncertain MDP, can date back to the 1970s [18–20]. There are many uncertainty models to describe the uncertain transition probabilities. The most common approach is to assume that the uncertain transition probabilities lie in an interval [13, 21]. The interval model is motivated by statistical estimates of confidence intervals on the individual components of the transition probability. Other statistic uncertainty models, which have been explicitly described in [22], include the likelihood model, the Bayesian model, and the entropy model. Relevant algorithms have been developed to handle uncertain MDPs, which mainly employ the game theory, the queuing theory, or dynamic programming. The most commonly used method is the min-max robust decision-making method [22], where the principle of optimality criterion is to maximize the worst-case expected total utility. Based on this optimality criterion, robust value iteration and robust policy iteration [23, 24] algorithms are proposed to obtain a min-max robust policy, and they both depend on robust dynamic programming, which may address the issue of designing an approximation method with an appropriate robustness to extend the power of the Bellman Equation. Iyengar [25] and Nilim and El Ghaoui [22] suggest to find a policy to guarantee the highest expected total utility at a given confidence level. To this end, a policy that maximizes the worst-case objective is determined. Bertuccelli et al [26] develop a robust adaptive MDP to optimize the UAV decisions. Although a great deal of researches have been done, robust decision making for uncertain MDPs with LTL specifications is rarely considered. To our knowledge, there are only a few studies on this problem. Wolff et al [27] propose a robust version of dynamic programming to work out a min-max robust policy, which maximizes the worst-case probability of satisfying LTL specifications. The previous robust methods are disadvantageous in several aspects. First, the uncertainty models require adequate knowledge of the uncertainty range (e.g., the interval model) or probabilistic information (e.g., Bayesian and likelihood models) in advance. It cannot handle the situation where the uncertainty is fuzzy, non-probabilistic or unknown, which is called severe uncertainty [28]. Since severe uncertainty often occurs in uncertain and dynamic environments [29], UAVs have to make robust decisions in order to reduce the influence of severe uncertainty. Second, the min-max robust decision-making method may lead to an overly conservative resulting policy, which indicates the policy will be sub-optimal in any case but the worst case. Furthermore, determining the worst case would become infeasible when the uncertainty is severe.

Motivated by the previous two limitations, another robust decision theory, namely the info-gap decision theory [28], is proposed to solve the robust decision-making problem from another perspective, which has been applied in a large spectrum of fields, including engineering, biology, and project management. It is a conceptual framework that can support making decisions under severe uncertainty and that has substantive implications for the formulation, evaluation and selection of desired goals and of the means to attain them. First, the uncertainty model is non-probabilistic, defined by a family of nested sets. That is, it does not require one to specify either a probability distribution or bounds on the uncertainty set. Second, the info-gap decision theory derives a robust satisficing policy by maximizing the robustness (i.e., the immunity to uncertainty) while satisfying a desired performance level, instead of seeking for the optimal utility. The term ‘satisficing’, a combination of satisfy and suffice [30], is introduced by Simon in 1956 [31]. Satisficing is a decision-making strategy aimed at a satisfactory or adequate result, rather than the optimal solution. For our problem, when the UAV executes complex civil or military missions, human operators often specify some critical mission criterions instead of the optimal ones, because the optimality is often difficult to attain or costly in terms of pay in real applications. Moreover, the robustness is a reliable principle often used in complex decision-making problems involving severe uncertainty [32, 33] because it provides the maximum reduction of unknown risks. Therefore, we will propose a robust satisficing decision-making method based on the info-gap decision theory to solve the robust decision-making problem for the UAV executing complex missions under uncertainty, i.e., the synthesizing control of uncertain MDPs with LTL specifications. The goal is to generate a robust satisficing policy that can maximize the robustness while ensuring the desired probability level of satisfying the LTL specifications. There are several works using the info-gap decision theory to obtain a robust satisficing policy for their research areas, such as neural network [34], multiagent search [35], and path planning [36]. S. Gareth et al [34] use an info-gap model to quantify the network response to uncertainty in the input data in order to evaluate the reliability of the neural network. Itay et al [35] propose a robust satisficing approach based on the info-gap decision theory to solve the spatial search-planning problem with imprecise probabilistic data. Mascareñas et al [36] develop a path planner anchored in the info-gap decision theory to generate non-deterministic paths that satisfy predetermined performance requirements in the face of the uncertain actions of the hostile elements. However, all the previous works do not consider the system model as a MDP and the complex mission requirements, even with regard to the synthesizing control problem of uncertain MDPs with LTL specifications. Therefore, utilizing the info-gap decision theory to solve robust UAV decision making for complex missions under severe uncertainty is still an open problem.

Our work is carried out based on the combination of the MDP and the info-gap decision theory. The LTL is utilized to describe the complex mission requirements of UAVs, which is introduced as constraints of the optimization problem. The main contribution of this paper is as follows. A robust satisficing decision-making method based on the info-gap decision theory is proposed for the robust UAVs decision-making problem with complex mission requirements under severe uncertainty. First, motivated by the info-gap decision theory, we propose a novel optimization problem for the robust UAVs decision-making problem, which can maximize the robustness while ensuring the desired mission requirements. Specifically, the uncertain UAV system is modeled as a new info-gap based MDP, and a robustness function is formulated to evaluate the robustness with the LTL formula specifying the mission specifications, so a robust satisficing policy is obtained to achieve the maximal robustness. To our knowledge, this is the first work that extends the robust satisficing concept into UAV robust decision making under severe uncertainty, with consideration given to critical mission specifications. Second, a two-stage robust satisficing solution strategy is proposed to solve the previous problem, which consists of the construction of a product IMDP and the generation of a robust satisficing policy. In the first stage, the product IMDP is constructed by creating the Cartesian product of the IMDP and DRA converted from the LTL formula, which is used to compute the probability of satisfying the LTL formula. In the second stage: i) the monotonic relationship between the uncertainty level and the worst-case probability of satisfying the LTL formula is provided and proven, so that when the uncertainty level continuously increases, the worst-case probability will reach the critical satisfying condition of the desired performance level; ii) based on the previous monotonic relationship, the robust satisficing optimality theorem is proved, which can help improve the policy towards the direction with a higher robustness; and iii) an algorithm based on robust dynamic programming is proposed to generate a robust satisficing policy, while an associated robustness evaluation algorithm is presented to evaluate the robustness. Finally, through Monte Carlo simulation, the effectiveness of our algorithm is demonstrated on an UAV search mission under severe uncertainty so that the resulting policy can maximize the robustness while satisfying the desired performance level. Further, by comparing the proposed method with the min-max robust decision-making method and the robust decision making [37](another robust optimization method for handling the severe uncertainty), it can be concluded that our policy can tolerate higher uncertainty so that the desired performance level can be guaranteed, which indicates that the proposed method is much more effective in real applications of the UAVs decision making problem.

The remainder of this paper is organized as follows. Some preliminary definitions are presented in Section 2. The problem is formulated based on the info-gap decision theory and the solution method is outlined in Section 3. In Section 4, a robust satisficing policy generation algorithm based on robust dynamic programming is proposed as well as a robustness evaluation algorithm. In Section 5, our algorithms are illustrated by an example of the UAV search mission, and the results are analyzed. The conclusion and future work are presented in Section 6.

## 2 Preliminaries

In this section, we will present some preliminary definitions that will be used in this paper.

### 2.1 LTL Specifications

The LTL formula is used to specify the complex mission with temporal constraints.

**Definition 1 (LTL** [14]**)**: An LTL formula *ϕ* can be defined recursively by a set of atomic propositions *AP* and a set of unary and binary operators:

ϕ⩴p|¬p|ϕ∨ϕ|ϕ∧ϕ|Xϕ|ϕUϕ|Fϕ|Gϕ

where *p* ∈ *AP* is an atomic proposition; ¬ (negation), ∨ (disjunction), and ∧ (conjunction) are the standard Boolean operators; *X* (next), *U* (until), *F* (eventually), and *G* (always) are the temporal operators.

The semantics of LTL formulas are defined over infinite words in 2^*AP*^. Given an infinite word *σ* = *τ*_0_*τ*_1_ … *τ*_*i*_ …, if the LTL formula *ϕ* is true at the first position, we say the word satisfies the LTL formula *ϕ*, denoted as *σ* ⊨ *ϕ*. *Xϕ* means that *ϕ* becomes true at the next position of the word; *Gϕ* means that *ϕ* is true at all positions of the word; *Fϕ* means that *ϕ* eventually becomes true in the word; *ϕ*_1_*Uϕ*_2_ means that *ϕ*_1_ holds at each position in the word until *ϕ*_2_ is true.

In quantitative probabilistic verification, an LTL formula needs to be translated into a deterministic Rabin automaton (DRA) by the PRISM [38], a leading probabilistic model checker.

**Definition 2 (DRA** [14]**)**: A DRA is a tuple *A*_*ϕ*_ = {*Q*, *q*_0_, Σ, *δ*, *Acc*}, where *Q* is a finite set of states, *q*_0_ ∈ *Q* is the initial state, Σ = 2^*AP*^ is an input alphabet, *δ*: *Q* × Σ → *Q* is the transition function, and *Acc* ⊆ 2^*Q*^ × 2^*Q*^ is a set of accepting state pairs.

Let *ω* = *ω*_0_*ω*_1_ … be a string over Σ. A run *ω* denotes an infinite sequence *q*_0_*q*_1_ … of states in *A*_*ϕ*_ such that *q*_*i*+1_ = *δ*(*q*_*i*_, *ω*_*i*_) for *i* ≥ 0. The run is accepted by a DRA, if for an accepting pair (*J*, *K*) ∈ *Acc*, the set of states *J* is finitely often visited and the set of states *K* is infinitely often visited.

### 2.2 System Model

We consider the UAV system with noisy actuation of which the dynamics are described by a stochastic differential equation [13]. It is assumed that the evolution of the stochastic system satisfies the Markov property, which can be abstracted to an MDP.

**Definition 3 (MDP** [12]**)**: A (labeled) MDP is defined as a tuple <*S*, *A*, *P*, *R*, *s*_0_, *AP*, *L*>, where *S* is a finite set of states; *A* is a finite set of actions (*A*(*s*) ⊆ *A* denotes the actions available at state *s* ∈ *S*); *P*: *S* × *A* × *S* → [0, 1] is the transition probability function, such that for all *s* ∈ *S*, ∑_*s*′∈*S*_ *P*(*s*, *a*, *s*′) = 1 if *a* ∈ *A*(*s*), and *P*(*s*, *a*, *s*′) = 0 if *a* ∉ *A*(*s*); $R:S\times A\to R^{+}$ is the reward function; *s*_0_ ∈ *S* is the initial state; *AP* is a finite set of atomic propositions; and *L*: *S* → 2^*AP*^ is a labeling function that establishes which atomic propositions are true in the given state *s* ∈ *S*, i.e., *L* relates discrete states with the proposition regions.

We use $P_{ij}^{a}$ as shorthand for the transition probability from state *i* to state *j* when using action *a*. *P*^*a*^: *S* × *S* → [0, 1] represents a transition matrix, where the (*i*, *j*)-th entry of *P*^*a*^ is $P_{ij}^{a}$. When the transition probabilities of the MDP are uncertain, it is only known that the corresponding transition matrix *P*^*a*^ for each action *a* lies in some given subset $P^{a}$.

**Definition 4 (Uncertain MDP** [22]**)**: The uncertain MDP is defined as $uM=<S,A,P,R,s_{0},AP,L>$, where $P=P^{1}\times \ldots \times P^{\left|A\right|}$ is the uncertainty set for the transition probabilities. For all *a* ∈ *A*, it is assumed that the sets $P^{a}$ satisfy the rectangular uncertainty property, i.e., $P^{a}=P_{1}^{a}\times \ldots \times P_{n}^{a}$.

### 2.3 Info-gap Decision Theory

The info-gap decision theory consists of three components: an info-gap uncertainty model, a robustness function, and a robust satisficing policy.

**Definition 5 (Info-gap uncertainty model** [28]**)**: Assuming the best estimation of an uncertain parameter *u* is $\tilde{u}$, and the relative errors between these two values are unknown, the info-gap uncertainty model can be represented as a family of nested sets:

$$U\left(\alpha ,\tilde{u}\right)=\{u:|u-\tilde{u}\left|\leq \alpha \tilde{u}\right\},\alpha \geq 0$$ (1)

where *α* is the unknown fractional deviation from the estimated value, i.e. the uncertainty level. In this paper, it is assumed that $\tilde{u}$ is the estimated transition probability of the MDP, which will be explicitly described in the next subsection.

**Fig 1:**
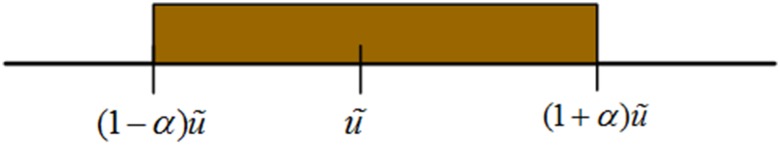
The space of possible values of uncertain variables.

The set $U(\alpha ,\tilde{u})$ contains all parameters *u* of which fractional deviation from $\tilde{u}$ is no greater than *α*, as shown in Fig 1.

The info-gap uncertainty model obeys two axioms:

- Contraction: $U\left(0,\tilde{u}\right)=\left\{\tilde{u}\right\}$.
- Nesting: $\alpha_{1}\leq \alpha_{2}\Rightarrow U\left(\alpha_{1},\tilde{u}\right)\subseteq U(\alpha_{1},\tilde{u})$.

From the contraction property, we can see that $\tilde{u}$ is the only value if there is no uncertainty. And the second property states that the higher the uncertainty level is, the more inclusive the info-gap uncertainty model will be.

The robustness function of the info-gap decision theory measures the highest uncertainty level for which a given policy will satisfy the performance requirements, thus allowing maximal lack in the knowledge of a priori information.

**Definition 6 (Robustness function** [28]**)**: In the info-gap theory, the robustness function of a given policy *π* is defined as the highest level of uncertainty that can be tolerated, for which the given policy will satisfy a desired performance level *r*_*c*_

$$\hat{\alpha }\left(\pi ,r_{c}\right)=\max\left\{\alpha :\min_{u\in U(\alpha ,\tilde{u})}R\left(\pi ,u\right)\geq r_{c}\right\}$$ (2)

where $U(\alpha ,\tilde{u})$ is the info-gap uncertainty model, and *R*(*π*, *u*) is the performance evaluation function for policy *π* and uncertain parameter *u*.

**Definition 7 (Robust satisficing policy** [28]**)**: A robust satisficing policy is defined as the policy that maximizes the robustness function Eq (2) while satisfying the desired performance level *r*_*c*_

$$\pi^{*}\left(r_{c}\right)=\arg\max_{\pi \in \Pi }\hat{\alpha }\left(\pi ,r_{c}\right)$$ (3)

where Π represents the decision space that consists of a set of possible policies.

The robust satisficing policy π*(*r*_*c*_) maximizes $\hat{\alpha }\left(\pi ,r_{c}\right)$ conditional on the desired performance level *r*_*c*_, and makes the condition *R*(π*(*r*_*c*_), *u*) ≥ *r*_*c*_ guaranteed for any $u\in U(\alpha ,\tilde{u})$.

## 3 Info-gap Based Robust Satisficing Decision-Making Problem

### 3.1 Info-gap based MDP

In this paper, we use the info-gap uncertainty model to represent the uncertain transition probabilities of the MDP. Let *P* be the unknown true transition probability, $\tilde{P}$ be the estimated transition probability of the MDP, and *P*^*a*^ and ${\tilde{P}}^{a}$ be the transition matrices of *P* and $\tilde{P}$ for taking action *a* respectively. The info-gap uncertainty model of the transition matrix for taking action *a* is defined as follows

$$U^{a}\left(\alpha ,\tilde{P}\right)=\left\{P^{a}:\left|p_{i}-{\tilde{p}}_{i}\right|\leq \alpha {\tilde{p}}_{i},\;0\leq \alpha \leq 1,\;p_{i}1=1,\;{\tilde{p}}_{i}1=1,\;p_{i}\geq 0,\;\tilde{p_{i}}\geq 0\right\}\;\forall i$$ (4)

where *p*_*i*_ and ${\tilde{p}}_{i}$ represent the *i*th row of *P*^*a*^ and ${\tilde{P}}^{a}$, and *α* is the unknown uncertainty level. The uncertainty set of *p*_*i*_ can be expressed as an interval $\left[\underline{p},\bar{p}\right]$, where $\underline{p}=\left(1-\alpha \right)\tilde{p}$, $\bar{p}=\left(1+\alpha \right)\tilde{p}$, and $\underline{p},\bar{p}\geq 0$. Since *α* is unknown, the interval model is not fixed. The range of uncertainty expands as *α* increases.

**Definition 8 (IMDP)**: The info-gap based (labeled) MDP (IMDP) is defined as a tuple $IM=<S,A,U\left(\alpha ,\tilde{P}\right),R,s_{0},AP,L>$ by replacing P in the uncertain MDP with *U*, where $U\left(\alpha ,\tilde{P}\right)=U^{1}\left(\alpha ,{\tilde{P}}^{1}\right)\times \ldots \times U^{\left|A\right|}\left(\alpha ,{\tilde{P}}^{\left|A\right|}\right)$ are the sets of all possible transition matrices, as defined in Eq (4).

A control policy for the IMDP is defined as a sequence *π* = {*μ*_0_, *μ*_1_, …}, where *μ*_*i*_: *S* → *A* is a control function such that *μ*(*s*) ∈ *A*(*s*) for all *s* ∈ *S*. If *π* = {*μ*, *μ*, …}, the control policy is called a stationary policy.

### 3.2 Problem Formulation

Considering both the robustness of an IMDP model and the probability of satisfying the LTL specifications, we propose an info-gap based robust satisficing decision-making method. The objective is to maximize the robustness to uncertainty in the IMDP model while guaranteeing the desired performance level satisfied. In this paper, the performance level is defined as the desired mission success rate of the UAV, i.e., the desired probability level of satisfying the LTL specifications.

**Definition 9 (LSP and DLSP)**: The probability of satisfying the LTL specification *ϕ* (LSP) by the IMDP *IM* under the control policy *π* is defined as *Pr*^*π*^(*s*_0_ ⊨ *π*). And the desired probability level of satisfying the LTL specification (DLSP) is set as a constant value within (0, 1) by the UAV operator.

A control policy *π* of the IMDP *IM* can produce a path $r_{IM}^{\pi }=s_{0}s_{1}\ldots s_{i}\ldots$ over *IM*, which will further generate a corresponding infinite word *σ* = *τ*_0_*τ*_1_ … *τ*_*i*_ … over the atomic propositions of the LTL such that *τ*_*i*_ = *L*(*s*_*i*_). Thus, the probability of satisfying the LTL formula *ϕ* (LSP) for a path produced by policy *ϕ* over *IM* can be represented as $Pr^{\pi }\left(s_{0}⊧\pi \right)=Pr\left\{L\left(r_{IM}^{\pi }\right)⊧\phi \right\}$, which is measurable [14].

Formally, the robust satisficing decision-making problem can be formulated as follows:

**Problem 1**: Let $IM=<S,A,U\left(\alpha ,\tilde{P}\right),R,\gamma ,s_{0},L,AP>$ be an IMDP, and *ϕ* be an LTL formula over *AP*. The objective is to generate a robust satisficing policy π*(*p*_*c*_) that maximizes the robustness while guaranteeing the DLSP *p*_*c*_ satisfied

$$\pi^{*}\left(p_{c}\right)=\arg\max_{\pi \in \Pi }\alpha$$ (5)

$$s.t.\min_{P\in U(\alpha ,\tilde{P})}Pr^{\pi }\left(s_{0}⊧\phi \right)\geq p_{c}$$ (6)

where Π represents the decision space consisting of a set of possible policies, and *Pr*^*π*^(*s*_0_ ⊨ *ϕ*) is defined as the LSP of the LTL formula *ϕ* by *IM* under the control policy *π* from an initial state *s*_0_.

For a DLSP *p*_*c*_, the robustness of a policy *π* can be defined as

$$\hat{\alpha }\left(\pi ,p_{c}\right)=\max\alpha$$ (7)

$$s.t.\min_{P\in U(\alpha ,\tilde{P})}Pr^{\pi }\left(s_{0}⊧\phi \right)\geq p_{c}$$ (8)

**Remark 1**: For a set of feasible policies, the preference can be determined by the robustness of each policy. The higher the robustness is, the higher the preference will be.

### 3.3 Robust Satisficing Decision-Making Framework

**Fig 2:**
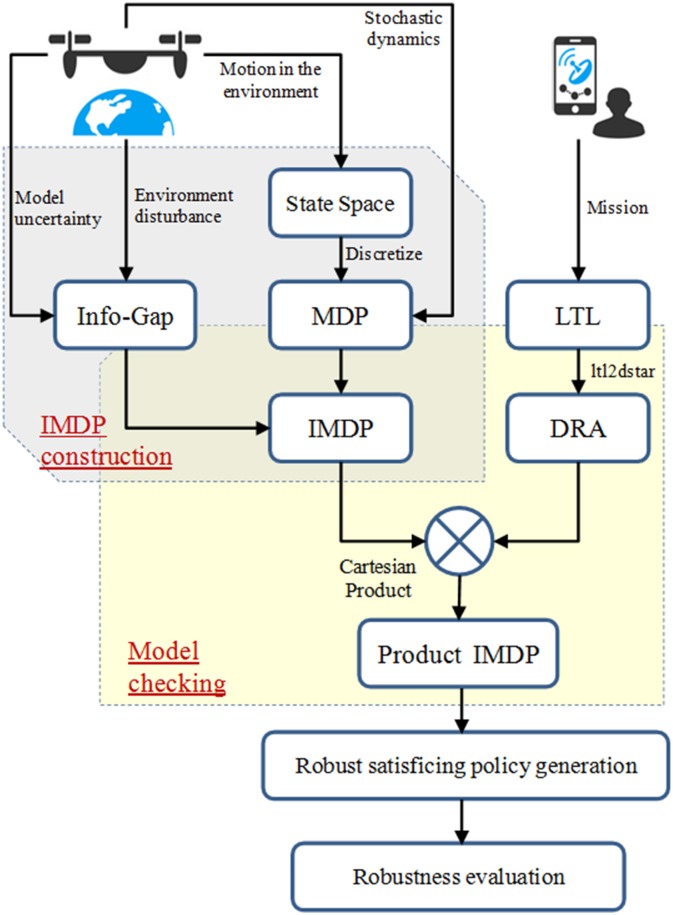
Robust satisficing decision-making framework.

In this subsection, we will present a robust satisficing decision-making framework for Problem 1, which is a computational framework that produces a policy that maximizes the robustness to uncertainty while guaranteeing the DLSP satisfied, as shown in Fig 2. This framework consists of two main parts, the construction of the product IMDP and the generation of a robust satisficing policy, which will be described in detail in the following.

#### 3.3.1 Construction of the Product IMDP

According to the model checking theory [14], the LSP for the IMDP is equivalent to the probability of reaching an accepting maximal end component (AMEC) of the product IMDP for a given policy and a given transition probability. By constructing the product IMDP, the LSP can be computed. Therefore, we start by constructing the product IMDP, which is used for computing the LSP in order to determine whether the DLSP in Problem 1 is satisfied or not. First, the LTL formula should be converted into a DRA as defined in Def. 2. The product IMDP is constructed by creating the Cartesian product of the IMDP and DRA.

**Definition 10 (Product IMDP)**: Let $IM=<S,A,U\left(\alpha ,\tilde{P}\right),R,s_{0},AP,L>$ be an IMDP. The LTL formula is converted into a DRA as *A*_*ϕ*_ = {*Q*, *q*_0_, Σ, *δ*, *Acc*}. The product IMDP is defined by $P=<S_{P},A_{P},U_{P}\left(\alpha ,\tilde{P}\right),R_{P},s_{P0},L_{P},Acc_{P}>$, where

- *S*_*P*_ = *S* × *Q*.
- *A*_*P*_(*s*, *q*) = *A*(*s*).
- For $P_{P}\in U_{P}\left(\alpha ,\tilde{P}\right)$ and $P\in U(\alpha ,\tilde{P})$, *P*_*P*_((*s*, *q*), *a*, (*s*′, *q*′)) = *P*(*s*, *a*, *s*′) if *q*′ = *δ*(*q*, *L*(*s*)); otherwise, 0.
- *R*_*P*_((*s*, *q*), *a*) = *R*(*s*, *a*).
- *s*_*P*0_ = (*s*_0_, *q*_0_).
- *L*_*P*_(*s*, *q*) = *q*.
- $Acc_{P}=\left\{\left(J_{1}^{P},K_{1}^{P}\right),\left(J_{2}^{P},K_{2}^{P}\right),\ldots \right\}$. For (*L*_*i*_, *J*_*i*_) ∈ *Acc*, state $\left(s,q\right)\in J_{i}^{P}$ if *q* ∈ *J*_*i*_, and state $\left(s,q\right)\in L_{i}^{P}$ if *q* ∈ *L*_*i*_.

The policy $\pi^{P}=\left\{\mu_{0}^{P},\mu_{1}^{P},\ldots \right\}$ on the product IMDP is denoted as $\mu_{i}^{P}:S\times Q\to A$. There is a one-to-one correspondence between the paths on the IMDP and the product IMDP, which induces a one-to-one correspondence between the policies on the IMDP and the product IMDP. Therefore, given a policy $\pi^{P}=\left\{\mu_{0}^{P},\mu_{1}^{P},\ldots \right\}$ on the product IMDP, one can induce a policy *π* = {*μ*_0_, *μ*_1_, …} on the IMDP by setting $\mu_{i}^{P}\left(s,q\right)=\mu_{i}\left(s\right)$ for *i* = 0, 1, ….

With the product IMDP, the detailed procedure of obtaining the AMECs is outlined in [14].

**Definition 11 (AMEC** [14]**)**: The accepting maximal end component is defined as $\left({\bar{S}}_{P},{\bar{A}}_{P}\right)$, consisting of a set of states ${\bar{S}}_{P}\subseteq S_{P}$ and a function ${\bar{A}}_{P}\left(s_{P}\right)\subseteq A_{P}\left(s_{P}\right)$, which implies that by taking actions enabled by ${\bar{A}}_{P}$, all states in ${\bar{S}}_{P}$ can reach every other state in ${\bar{S}}_{P}$ and cannot reach any state outside of ${\bar{S}}_{P}$.

Once an AMEC is reached, all states in ${\bar{S}}_{P}$ are infinitely often reached with probability 1, by taking all actions in ${\bar{A}}_{P}$. The LSP is the maximum probability of reaching any states in ${\bar{S}}_{P}$ from an initial state *s*_*P*0_ ∈ *S*_*P*_. We can find the set of states that can never reach ${\bar{S}}_{P}$ under any policy via the graph theory, denoted as *B*_0_. The set of the rest states is $B_{P}=S_{P}/\left({\bar{S}}_{P}⋃B_{0}\right)$. According to the model checking theory, the LSP from the initial state *s*_*P*_ can be determined as: 1 if $s_{P}\in {\bar{S}}_{P}$, or 0 if *s*_*P*_ ∈ *B*_0_. For *s*_*P*_ ∈ *B*_*P*_, the LSP can be obtained through linear or dynamic programming [14] [11] if there is no uncertainty. However, in this paper the system is abstracted into the IMDP model, and the transition probability lies in an info-gap uncertainty model, which requires the robust satisficing solution method. In the next subsection, the solution scheme will be presented for obtaining a robust satisficing policy for Problem 1.

#### 3.3.2 Solution Scheme

**Fig 3:**
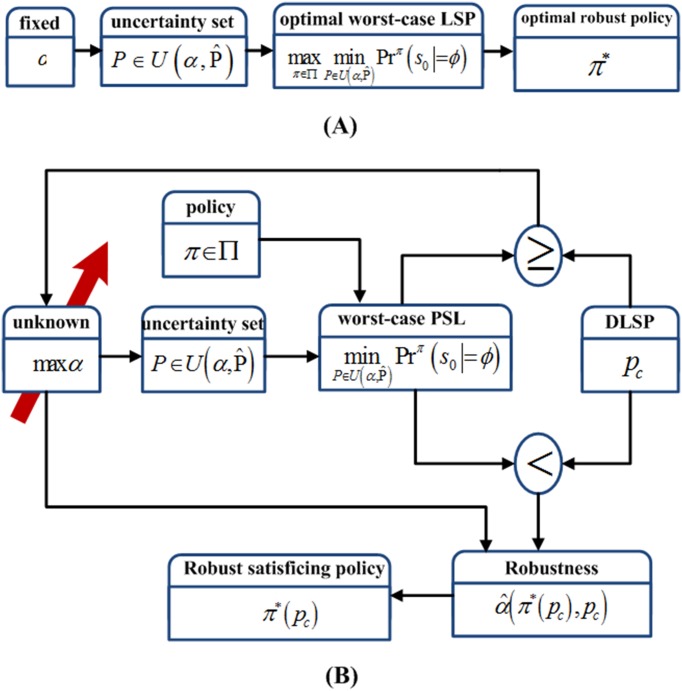
The solution schemes for the min-max robust decision-making method and the robust satisficing decision-making method. (A) Solution scheme for the min-max robust decision-making method. (B) Solution scheme for the robust satisficing decision-making method.

It can be seen that Problem 1 for finding the robust satisficing policy is essentially a complex optimization problem. Both the policy *π* and the uncertainty level *α* will affect the value of the worst-case LSP, so they will affect determining whether the DLSP is satisfied or not. In order to clarify the solution procedure of generating a robust satisficing policy, it is compared with that of the min-max decision-making method. The solution schemes are shown in Fig 3.

Considering the IMDP model and the LTL specification, the min-max robust decision-making method is to create an optimal robust policy to maximize the worst-case LSP at a specified uncertainty level $\bar{\alpha }$. It can be regarded as a game between the environment and the decision maker, and the optimal robust policy is

$$\pi^{*}=\arg\max_{\pi \in \Pi }\min_{P\in U(\bar{\alpha },\tilde{P})}Pr^{\pi }\left(s_{0}⊧\phi \right)$$ (9)

This min-max optimization problem written as Eq (9) can be solved to find an optimal robust policy through robust dynamic programming. The solution scheme can be seen in Fig 3(A). However, it is only feasible when the uncertain transition probability set is fixed, i.e., the uncertainty level $\bar{\alpha }$ is known in advance.

The solution scheme for Problem 1, i.e., the robust satisficing decision-making method, is shown in Fig 3(B). In order to simplify the computation, the uncertainty level within [0,1] will be divided into N uniform divisions. We start by choosing the uncertainty level *α*_*t*_ as zero. For each specified uncertainty level *α*_*t*_, a policy with the highest worst-case LSP will be found, and the highest worst-case LSP will be compared with the DLSP. If the highest worst-case LSP is higher than or equal to the DLSP, the value of the uncertainty level will be increased by *α*_*t*+1_ = *α*_*t*_+1/*N*, and the above procedures will be repeated; otherwise, the last policy with the highest worst-case value is the robust satisficing policy, and its corresponding uncertainty level is its robustness for the DLSP.

The solution scheme is designed based on several lemmas and theorems which will be provided in next section. The solution procedure for Problem 1 can be outlined as follows:

1. The monotonic relationship between the uncertainty level and the worst-case LSP will be established (Lemmas 1 and 2), which supports the division scheme of the uncertainty level. As the uncertainty level increases, the worst-case LSP will decrease such that a critical value of satisfying the DLSP will be reached.
2. The robust satisficing optimality theorems are provided and proved (Theorems 1 and 2), which will help improve the policy towards a higher robustness. A trade-off relationship between the robustness and the DLSP is also proved (Theorem 3).
3. The highest worst-case LSP is calculated as well as the corresponding optimal robust policy based on robust dynamic programming, which will be compared with the DLSP to determine whether the uncertainty level increases or not. And the robust satisficing policy generation algorithm is presented (Algorithm 1).
4. Furthermore, a robustness evaluation algorithm is presented, which can be used to evaluate the robustness of a fixed policy for a given DLSP (Algorithm 2).

## 4 Solution Method

In this section, we will explain each step of the solution procedure for Problem 1 in detail.

First, we begin with a simple claim. It is obvious that the choice of the DLSP will influence the robustness. Thus, a proper DLSP is determined such that a robust satisficing policy with nonzero robustness can be generated.

Let $V_{\pi }^{*}\left(s_{P},\alpha \right)=\min_{P_{P}\in U_{P}\left(\alpha ,\tilde{P}\right)}Pr^{\pi }$ denote the worst-case LSP of a given policy *π* starting from *s*_*P*_ at the uncertainty level *α*.

**Claim 1**: If the given DLSP *p*_*c*_ is higher than the LSP of the optimal policy without considering uncertainty, the robustness of all the policies for this DLSP will be zero.

**Proof**: The proof will be provided by contradiction. First, we assume that *π*_1_ is the optimal policy without considering uncertainty, and the worst case LSP of *π*_1_ is $V_{\pi_{1}}^{*}\left(s_{P},0\right)<p_{c}$. Assuming that there is an arbitrary policy *π*_2_ with robustness *α*_2_ ≠ 0 for the DLSP *p*_*c*_, we have

$$V_{\pi_{2}}^{*}\left(s_{P},\alpha_{2}\right)=\min_{P\in U(\alpha_{2},\tilde{P})}Pr^{\pi_{2}}\geq p_{c}$$ (10)

According to the assumption of the optimal policy *π*_1_, the following inequalities can be established

$$V_{\pi_{2}}^{*}\left(s_{P},\alpha_{2}\right)=\min_{P\in U(\alpha_{2},\tilde{P})}Pr^{\pi_{2}}\leq \min_{P\in U(0,\tilde{P})}Pr^{\pi_{2}}\leq \min_{P\in U(0,\tilde{P})}Pr^{\pi_{1}}=V_{\pi_{1}}^{*}\left(s_{P},0\right)<p_{c}$$ (11)

It’s obvious that the relationship of Eq (11) contradicts the assumption in Eq (10). Thus, the arbitrary policy *π*_2_ with non-zero robustness *π*_2_ does not exist.

According to Claim 1, if the given DLSP is higher than the LSP of the optimal policy without uncertainty, there would be a robust satisficing policy. In this case, the given DLSP cannot be satisfied by any policy, which means the mission specifications cannot be met. Therefore, if we would like to generate a robust satisficing policy, the desired satisfaction probability must be proper. Besides, as defined in the info-gap uncertainty model (4), the maximum robustness lies in [0, 1], which corresponds to a robust satisficing policy with the proper DLSP satisfied. In the subsequent section, it is assumed that the given DLSP is proper, i.e., not higher than the LSP of the optimal policy without considering uncertainty.

**Remark 2**: For a given proper DLSP, the maximum robustness Eq (5) will be achieved by the robust satisficing policy, i.e., $\hat{\alpha }\left(\pi^{*}\left(p_{c}\right),p_{c}\right)$. The robust satisficing policy π*(*p*_*c*_) Eq (7) maximizes $\hat{\alpha }\left(\pi ,p_{c}\right)$, which is conditional on some *p*_*c*_, and makes the condition *Pr*^π*(*p*_*c*_)^(*s*_0_ ⊨ *ϕ*) ≥ *p*_*c*_ guaranteed for any $P\in U(\hat{\alpha },\tilde{P})$.

### 4.1 Monotonicity

The monotonic relationship between the uncertainty level and the worst-case LSP of a given policy is presented.

**Lemma 1 (Monotonicity 1)**: For a given policy *π*, the worst-case LSP does not increase along with the uncertainty level, i.e., if *α*_1_ < *α*_2_, then $V_{\pi }^{*}\left(s_{P},\alpha_{1}\right)\geq V_{\pi }^{*}\left(s_{P},\alpha_{2}\right)$.

**Proof**: If the uncertainty level increases from *α*_1_ to *α*_2_, by the nesting axiom, the uncertainty set $U_{P}\left(\alpha_{2},\tilde{P}\right)$ will contain $U_{P}\left(\alpha_{1},\tilde{P}\right)$, i.e., $U_{P}\left(\alpha_{1},\tilde{P}\right)\subset U_{P}\left(\alpha_{2},\tilde{P}\right)$. It is assumed that $U_{P}\left(\alpha_{2},\tilde{P}\right)=U_{P}\left(\alpha_{1},\tilde{P}\right)⋃\Omega$, and Ω is a nonempty set. So there will be a relationship of the worst-case LSP between *α*_1_ and *α*_2_ as follows:

$$\begin{array}{lll} V_{\pi }^{*}(s_{P},\alpha_{2}) & = & \underset{P_{P}\in U_{P}(\alpha_{2},\tilde{P})}{\text{min}}Pr^{\pi }=\underset{P_{P}\in U_{P}(\alpha_{1},\tilde{P}){\cup \Omega }^{}}{\text{min}}Pr^{\pi }\\ & = & \text{min}\{\underset{P_{P}\in U_{P}(\alpha_{1},\tilde{P})}{\text{min}}Pr^{\pi },\underset{P_{P}\in \Omega }{\text{min}}Pr^{\pi }\}\\ & \leq & \underset{P_{P}\in U_{P}(\alpha_{1},\tilde{P})}{\text{min}}Pr^{\pi }=V_{\pi }^{*}(s_{P},\alpha_{1}) \end{array}$$

It can be concluded that if *α*_1_ < *α*_2_, $V_{\pi }^{*}\left(s_{P},\alpha_{1}\right)\geq V_{\pi }^{*}\left(s_{P},\alpha_{2}\right)$.

According to Lemma 1, the worst-case LSP $V_{\pi }^{*}\left(s_{P0},\alpha \right)$ of a fixed policy monotonically decreases at the uncertainty level *α*. So as *α* increases, the worst-case LSP of a fixed policy decreases. If *α* increases to *α*_*m*_, such that $V_{\pi }^{*}\left(s_{P0},\alpha_{m}\right)\geq p_{c}$, and $V_{\pi }^{*}\left(s_{P0},\alpha_{m}+\varepsilon \right)<p_{c}$ (*ε* is an infinitely small value), then *α*_*m*_ is the robustness of the fixed policy that is sought for.

Second, the monotonic relationship between the uncertainty level and the highest worst-case LSP is established.

Let $H^{*}\left(s_{P},\alpha \right)=\max_{\pi \in \Pi }\min_{P_{P}\in U_{P}\left(\alpha ,\tilde{P}\right)}Pr^{\pi }$ denote the highest worst-case LSP at the uncertainty level *α* from the initial state *s*_*P*_, i.e., $H^{*}\left(s_{P},\alpha \right)=\max_{\pi \in \Pi }V_{\pi }^{*}\left(s_{P},\alpha \right)$. The policy with the highest worst-case LSP at a given uncertainty level is called the optimal robust policy at this uncertainty level, which is also referred to as the min-max robust policy in this paper.

**Lemma 2 (Monotonicity 2)**: For all the policies, the highest worst-case LSP decreases as the uncertainty level increases, i.e., if *α*_1_ < *α*_2_, then H*(*s*_*P*_, *α*_1_)>H*(*s*_*P*_, *α*_2_).

**Proof**: It is assumed that *π*_1_ is the optimal robust policy at the uncertainty level *α*_1_, *π*_2_ is the optimal robust policy at the uncertainty level *α*_2_, and *α*_1_ < *α*_2_. Since *π*_1_ is the optimal robust policy at the uncertainty level *α*_1_, we have $H^{*}\left(s_{P},\alpha_{1}\right)=V_{\pi_{1}}^{*}\left(s_{P},\alpha_{1}\right)>V_{\pi_{2}}^{*}\left(s_{P},\alpha_{1}\right)$. Because *α*_1_ < *α*_2_, we have $V_{\pi_{2}}^{*}\left(s_{P},\alpha_{1}\right)>V_{\pi_{2}}^{*}\left(s_{P},\alpha_{2}\right)=H^{*}\left(s_{P},\alpha_{2}\right)$ according to Lemma 1. It can be concluded that if *α*_1_ < *α*_2_, H*(*s*_*P*_, *α*_1_) > H*(*s*_*P*_, *α*_2_).

According to the monotonicity in Lemma 2, there will exist a maximal value of *α*_*m*_ such that H*(*s*_*P*0_, *α*_*m*_) ≥ *p*_*c*_ and H*(*s*_*P*0_, *α*_*m*_ + *ε*) < *p*_*c*_, where *ε* is an infinitely small value. This maximal value *α*_*m*_ is the robustness of the optimal robust policy for the DLSP, and the optimal robust policy corresponding to the uncertainty level *α*_*m*_ is the robust satisficing policy.

### 4.2 Robust Satisficing Optimality

In order to obtain a robust satisficing policy, the feasible policy should be improved towards the direction with higher robustness. Thus Theorems 1 and 2 are given and proved to define the direction with higher robustness. In Theorem 3, the trade-off relationship between the DLSP and the robustness will be proved.

**Theorem 1 (Robustness optimality)**: For a given DLSP *p*_*c*_, the policy which maximizes the worst-case LSP will lead to a robustness higher than or equal to that obtained by any other policy at the same uncertainty level.

**Proof**: It is assumed that the robustness of *π*_1_ for the desired level *p*_*c*_ is *α*_1_, and *π*_1_ cannot maximize the worst-case LSP. Thus policy *π*_1_ satisfies $V_{\pi_{1}}^{*}\left(s_{P0},\alpha_{1}\right)\geq p_{c}$, and $V_{\pi_{1}}^{*}\left(s_{P0},\alpha_{1}+\varepsilon \right)<p_{c}$, where *ε* is an infinitely small value.

Let *π*_2_ be the policy that can maximize the worst-case LSP at the uncertainty level *α*_1_. We have

$$V_{\pi_{2}}^{*}\left(s_{P0},\alpha_{1}\right)=\max_{\pi \in \Pi }\min_{P_{P}\in U_{P}\left(\alpha_{1},\tilde{P}\right)}Pr^{\pi }>\min_{P_{P}\in U_{P}\left(\alpha_{1},\tilde{P}\right)}Pr^{\pi_{1}}=V_{\pi_{1}}^{*}\left(s_{P0},\alpha_{1}\right)\geq p_{c}$$

First the critical case for *π*_2_ is considered. It is assumed that for the infinitely small value *ε*, $V_{\pi_{2}}^{*}\left(s_{P0},\alpha_{1}+\varepsilon \right)<p_{c}$. Then the robustness of *π*_2_ is also *α*_1_. However, in other cases, if $V_{\pi_{2}}^{*}\left(s_{P0},\alpha_{1}+\varepsilon \right)\geq p_{c}$, there would be *α*_2_ ≥ *α*_1_ + *ε*, such that $V_{\pi_{2}}^{*}\left(s_{P0},\alpha_{2}\right)\geq p_{c}$ and $V_{\pi_{2}}^{*}\left(s_{P0},\alpha_{2}+\varepsilon \right)<p_{c}$. In this case, the robustness of *π*_2_ is *α*_2_. Therefore, the robustness of policy *π*_2_ is higher than that of policy *π*_1_.

**Theorem 2 (Robust satisficing optimality)**: For a given DLSP *p*_*c*_, a min-max robust policy which can maximize the worst-case LSP at a certain uncertainty level (maximum robustness) can be found, so it is the robust satisficing policy.

**Proof**: Let π*(*p*_*c*_) be the robust satisficing policy with the maximum robustness $\alpha_{m}=\hat{\alpha }\left(\pi^{*}\left(p_{c}\right),p_{c}\right)$. We have $V_{\pi^{*}\left(p_{c}\right)}^{*}\left(s_{P0},\alpha_{m}\right)\geq p_{c}$ and $V_{\pi^{*}\left(p_{c}\right)}^{*}\left(s_{P0},\alpha_{m}+\varepsilon \right)<p_{c}$, where *ε* is an infinitely small value.

It is assumed that *π*_*mm*_ is the min-max robust policy at the uncertainty level *α*_*m*_ and $V_{\pi_{mm}}^{*}\left(s_{P},\alpha_{m}\right)$ is the optimal worst-case LSP, which satisfies

$$V_{\pi_{mm}}^{*}\left(s_{P0},\alpha_{m}\right)\geq V_{\pi^{*}\left(p_{c}\right)}^{*}\left(s_{P0},\alpha_{m}\right)\geq p_{c}$$

First, it is assumed that the inequality holds. According to the proof of Theorem 1, the robustness of *π*_*mm*_ is higher than that of π*(*p*_*c*_) in all cases but the critical case. Thus there will exist *α*_*n*_ > *α*_*m*_, such that $V_{\pi_{mm}}^{*}\left(s_{P0},\alpha_{n}\right)\geq p_{c}$ and $V_{\pi_{mm}}^{*}\left(s_{P0},\alpha_{n}+\varepsilon \right)<p_{c}$. However, this violates the definition of the robust satisficing policy about maximal robustness. So the inequality condition is false. Under both the critical case and equality conditions, the robustness of *π*_*mm*_ is equal to that of π*(*p*_*c*_). That is, the min-max robust policy at the uncertainty level *α*_*m*_ is the robust satisficing policy for the DLSP *p*_*c*_.

**Theorem 3 (Trade-off Theorem)**: If the DLSP decreases, the robustness of a given policy or a robust satisficing policy will be non-decreasing, i.e., if $p_{c}>p_{c}^{\prime }$, then $\hat{\alpha }\left(\pi ,p_{c}\right)\leq \hat{\alpha }\left(\pi ,p_{c}^{\prime }\right)$.

**Proof**: It is assumed that a feasible robustness set for a given policy *π* is denoted as

$$\Lambda \left(\pi ,p_{c}\right)=\left\{\alpha :\min_{P_{P}\in U_{P}\left(\alpha ,\tilde{P}\right)}Pr^{\pi }\geq p_{c}\right\}$$

Thus the robustness is $\hat{\alpha }\left(\pi ,p_{c}\right)=\max\Lambda \left(\pi ,p_{c}\right)$.

It is assumed that $p_{c}>p_{c}^{\prime }$, and the feasible robustness sets Λ(*π*, *p*_*c*_) and $\Lambda (\pi ,p_{c}^{\prime })$ are non-empty. Let *α*_1_ ∈ Λ(*π*, *p*_*c*_), which means that $\min_{P_{P}\in U_{P}\left(\alpha_{1},\tilde{P}\right)}Pr^{\pi }\geq p_{c}\geq p_{c}^{\prime }$. It can be concluded that $\alpha_{1}\in \Lambda (\pi ,p_{c}^{\prime })$. Thus we have $\Lambda \left(\pi ,p_{c}\right)\subseteq \Lambda (\pi ,p_{c}^{\prime })$. According to the definition, it follows that $\hat{\alpha }\left(\pi ,p_{c}\right)=\max\Lambda \left(\pi ,p_{c}\right)\leq \max\Lambda (\pi ,p_{c}^{\prime })=\hat{\alpha }\left(\pi ,p_{c}^{\prime }\right)$. The trade-off relationship has thus been proved.

Theorem 3 shows that the robustness is monotonically decreasing at the DLSP. In practice, it may help the decision maker to decide a proper desired performance level by taking the robustness to uncertainty into consideration in order to realize a trade-off between the desired performance level and the robustness.

### 4.3 Calculation of the Highest Worst-Case LSP

According to Theorems 1 and 2, for a given DLSP *p*_*c*_, the robust satisficing policy can be regarded as the min-max robust policy at a certain uncertainty level. So in this subsection, we will present the calculation method of the highest worst-case LSP as well as the min-max robust policy at a fixed uncertainty level.

**Lemma 3 (Robust Dynamic Programming** [22]**)**: For the robust control problem, the perfect duality holds:

$$\Phi =\max_{\pi \in \Pi }\min_{P\in U(\bar{\alpha },\tilde{P})}Pr^{\pi }\left(s_{0}⊧\phi \right)=\min_{P\in U(\bar{\alpha },\tilde{P})}\max_{\pi \in \Pi }Pr^{\pi }\left(s_{0}⊧\phi \right)$$ (12)

The optimal value is given by Φ = v*(*s*_0_), where *s*_0_ is the initial state. For the product IMDP, the value function is the unique limit value of the convergent vector sequence defined by

$$v_{k+1}\left(s_{P}\right)=\max_{a\in A_{P}}\min_{P_{P}\in U_{P}\left(\bar{\alpha },\tilde{P}\right)}{\left(P_{P}\right)}^{T}v_{k},\;\;k=1,2,...$$ (13)

The optimal min-max robust control policy is obtained as

$$\pi^{*}\left(s_{P}\right)=\arg\max_{a\in A_{P}}\min_{P_{P}\in U_{P}\left(\bar{\alpha },\tilde{P}\right)}{\left(P_{P}\right)}^{T}v^{*}$$ (14)

For the proof of the detailed processes and convergence of the min-max robust policy, the readers can refer to Theorem 3 in [22].

In our problem, the highest worst-case LSP H*(*s*_*P*_, *α*) for a fixed uncertainty level *α* can be defined as the unique limit value of the following convergent vector sequence based on Lemma 3 (robust dynamic programming)

$$H_{k+1}\left(s_{P},\alpha \right)=\max_{a\in A_{P}}\min_{P_{P}^{a}\in \left[\underline{p},\bar{p}\right]}{\left(P_{P}^{a}\right)}^{T}H_{k}\left(•,\alpha \right)$$ (15)

where *H*_*k*_(•, *α*) is the vector of *H*_*k*_(*s*_*P*_, *α*) for all *s*_*P*_ ∈ *S*_*P*_ at stage *k*, and $\underline{p}$ and $\bar{p}$ denote the uncertainty interval for the *i*th row of $P_{P}^{a}$. The initial value for *H*_*k*_(*s*_*P*_, *α*) is given as the LSP related with AMECs in Subsection 3.3.1. Then, the worst-case LSP can be obtained via value iteration, which is described explicitly in Algorithm 1. Note that during each iteration, an inner minimization for finding the optimal transition probability will be computed based on the dual linear programming, which provides the worst-case condition. Refer to S1 Appendix for detailed procedures.

With the highest worst-case LSP H*(*s*_*P*_, *α*_*m*_), the corresponding min-max robust policy π*(*s*_*P*_) can be obtained by setting

$$\pi^{*}\left(s_{P}\right)=\arg\max_{a\in A_{P}}\min_{P_{P}^{a}\in \left[\underline{p},\bar{p}\right]}{\left(P_{P}^{a}\right)}^{T}H^{*}\left(•,\alpha_{m}\right)$$ (16)

### 4.4 Robust Satisficing Policy Generation Algorithm

In this subsection, a robust satisficing policy generation algorithm based on robust dynamic programming is proposed, as shown in Algorithm 1. The uncertainty level *α* is divided between 0 and 1 by *N* uniform divisions. For a specified value of *α*, the range of transition probability can be determined, and the highest worst-case LSP H*(*s*_*P*_, *α*) can be calculated via value iteration, as well as the corresponding min-max robust policy π* (Lines 8–19). Then, the highest worst-case LSP is compared with the given DLSP, and if the satisfaction condition holds, we will increase the uncertainty level by 1/*N* and repeat the above steps until the highest worst-case LSP reaches the given DLSP (Lines 20–24). An approximation value α* of robustness with the accuracy of 1/*N* can be determined by H*(*s*_*P*0_, α*) ≥ *p*_*c*_ and H*(*s*_*P*0_, α* + 1/*N*) < *p*_*c*_.

**Algorithm1** Robust Satisficing Policy Generation

**Required**: product IMDP $P=<S_{P},A_{P},U_{P}\left(\alpha ,\tilde{P}\right),R_{P},s_{P0},L_{P},Acc_{P}>$

**Required**: the DLSP *p*_*c*_

**Ensure**: Robust satisficing policy π*

**Ensure**: Maximal Robustness α*

 ▷ *Step* 0: *Initialization*

 ▷ *Step* 0.1: *Generate AMECs*

1:  Generate $\left({\bar{S}}_{P},{\bar{A}}_{P}\right)$, *B*_0_, *B*_*P*_

 ▷ *Step* 0.2: *Initialize LSP*

2:   **for** $s_{P}\in {\bar{S}}_{P}$ **do**

3:    *H*(*s*_*P*_, *α*) = 1

4:   **end for**

5:   **for** *s*_*P*_ ∈ *B*_0_ **do**

6:    *H*(*s*_*P*_, *α*) = 0

7:   **end for**

 ▷ *Step* 1: *Generate the robust satisficing policy and the maximal robustness*

 ▷ *Step* 1.1: *Generate the min*-*max* *based* *robust* *policy*

8:  *α* ← 0

9:  Δ ← ∞

10:  **while**Δ ≥ *ε*

11:   **for** $s_{P}\in S_{P}\setminus {\bar{S}}_{P}⋃B_{0}$ **do**

12:    *Max*_*P*_ ← *H*(•, *α*)

13:    $H\left(s_{P},\alpha \right)=\max_{a\in A_{P}}\min_{P_{P}^{a}\in \left[\underline{p},\bar{p}\right]}{\left(P_{P}^{a}\right)}^{T}H\left(•,\alpha \right)$

14:    $\pi =\arg\max_{a\in A_{P}}\min_{P_{P}^{a}\in \left[\underline{p},\bar{p}\right]}{\left(P_{P}^{a}\right)}^{T}H\left(•,\alpha \right)$

15:    Δ = min(∥*H*(•, *α*) − *Max*_*P*_∥, Δ)

16:    **if** Δ ≤ *ε* **then**

17:     H*(*s*_*P*_, *α*) = *H*(*s*_*P*_, *α*)

18:    **end if**

19:   **end for**

 ▷ *Step* 1.2: *Update the robustness*

20:  **if** ∥H*(*s*_*P*_, *α*) − *p*_*c*_∥ ≥ 0 **do**

21:   π* ← *π*

22:   α* ← *α* + 1/*N*

23:   *α* ← *α* + 1/*N*

24:   **goto** line 9

25:  **end if**

26: **end while**

27: **return** α*, π*

In the following, the convergence of the above algorithm will be provided.

**Theorem 4 (Convergence)**: For Problem 1, a robust satisficing policy, which is generated by Algorithm 1, can converge to the maximal robustness with a given proper DLSP *p*_*c*_ satisfied.

**Proof**: First, according to Lemma 3, for a specified uncertainty level *α*, a min-max robust policy can converge to a unique optimal value function, i.e., the highest worst-case LSP. In Algorithm 1, we use the discretized method to divide the uncertainty level into *N* equally spaced values *α*_*t*_, *t* = 1, …, *N* between 0 and 1. For each *α*_*t*_, the robust policy, generated by the robust dynamic programming, can converge to the highest worst-case LSP H*(*s*_*P*_, *α*_*t*_).

**Fig 4:**
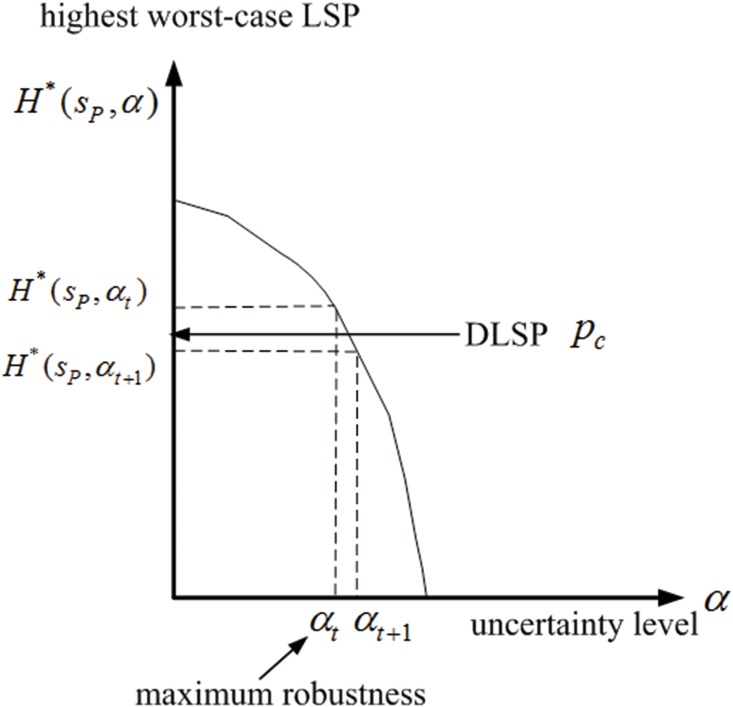
The monotonicity relationship between the highest worst-case LSP and the uncertainty level, and the maximum robustness.

Then, due to the monotonicity in Lemma 2, the highest worst-case LSP H*(*s*_*P*_, *α*) decreases as the uncertainty level *α* increases, as shown in Fig 4. Thus we can find a *α*_*t*_, such that H*(*s*_*P*_, *α*_*t*_) ≥ *p*_*c*_ and H*(*s*_*P*_, *α*_*t*+1_) < *p*_*c*_, where H*(*s*_*P*_, *α*_*t*_) can be achieved by the min-max policy $\pi_{mm}^{t}$ at the uncertainty level *α*_*t*_. In this case, *α*_*t*_ is the maximum robustness with the accuracy 1/*N*, as shown in Fig 4.

Finally, according to Theorem 2, the min-max robust policy $\pi_{mm}^{t}$ with the highest worst-case LSP H*(*s*_*P*_, *α*_*t*_) at *α*_*t*_ is the robust satisficing policy for the DLSP *p*_*c*_ with the maximum robustness *α*_*t*_.

**Remark 3**: The complexity of Algorithm 1 is affected by the size of the product IMDP. The size of the DRA |*Q*| is in the worst case, doubly exponential with respect to the LTL formula [39]. The size of the product IMDP is at least *n* = |*S*| × |*Q*|, without considering the uncertain transition matrices. For the uncertain IMDP, the complexity of solving the worst-case problem is *O*(*n*log(*n*)). And the maximal end components can be generated in *O*(*n*^2^) at most. Algorithm 1 spends at most *O*(*n*^2^*aN*(*log*(1/*ε*))^2^) to obtain the robust satisficing policy, where *N* is the number of robustness divisions and *a* = |*A*| is the number of actions.

### 4.5 Robustness Evaluation Algorithm

Algorithm 1 is used to generate a robust satisficing policy. However, if there are several feasible policies, a robustness evaluation algorithm is required to choose the policy with the highest robustness. The robustness evaluation problem, for evaluating the robustness of a fixed policy *π* for a given DLSP *p*_*c*_, can be formulated as

$$\hat{\alpha }\left(\pi ,p_{c}\right)=\max\left\{\alpha :\min_{P_{P}\in U_{P}\left(\alpha ,\tilde{P}\right)}Pr^{\pi }\left(s_{0}⊧\phi \right)\geq p_{c}\right\}=\max\left\{\alpha :V_{\pi }^{*}\left(s_{P0},\alpha \right)\geq p_{c}\right\}$$ (17)

The solution scheme of robustness evaluation is similar to that for generating the robust satisficing policy. The Bellman recursion will be used to calculate the worst-case LSP, which satisfies the following conditions

$$V_{\pi }^{*}\left(s_{P},\alpha \right)=\min_{P_{P}^{a}\in \left[\underline{p},\bar{p}\right]}P_{P}^{a}{\left(s_{P},s_{P}^{\prime }\right)}^{T}V_{\pi }^{*}\left(s_{P}^{\prime },\alpha \right),s_{p}\in S_{P}$$ (18)

where *a* = *π*(*s*_*P*_). $V_{\pi }^{*}\left(s_{P},\alpha \right)$ for policy *π* is the unique fixed point of the contraction mapping $\Phi \left(\pi ,s_{P}\right):R^{|S_{P}|}\to R^{|S_{P}|}$ defined by

$$\Phi \left(\pi ,s_{P}\right)=\min_{P_{P}^{a}\in \left[\underline{p},\bar{p}\right]}{\left(P_{P}^{a}\right)}^{T}V_{\pi }\left(•,\alpha \right)$$ (19)

where *V*_*π*_(•, *α*) is the vector of $V_{\pi }^{*}\left(s_{P},\alpha \right)$ for all *s*_*P*_ ∈ *S*_*P*_. For the proof of the contraction mapping, refer to Lemma 2 in [27]. The worst-case LSP, as the fixed point of the contraction mapping, can be obtained via value iteration. Then a robustness evaluation algorithm is proposed so as to find the robustness of a given policy for a given DLSP, as shown in Algorithm 2.

**Algorithm2** Robustness Evaluation for a Specified Policy

**Required**: product IMDP $P=<S_{P},A_{P},U_{P}\left(\alpha ,\tilde{P}\right),R_{P},s_{P0},L_{P},Acc_{P}>$

**Required**: a specified policy *π*

**Required**: the DLSP *p*_*c*_

**Ensure**: Robustness α*

 ▷ *Step* 0: *Initialization*

1:  Generate AMECs $\left({\bar{S}}_{P},{\bar{A}}_{P}\right)$, *B*_0_, *B*_*P*_

2:  for $s_{P}\in {\bar{S}}_{P}⋃B_{0}$, initialize *V*_*π*_(•, *α*)

3:  Δ ← ∞

4:  *α* ← 0

 ▷ *Step* 1: *Evaluate the robustness*

5:  **while** *V*_*π*_(*s*_*P*0_, *α*) − *p*_*c*_ ≥ 0

 ▷ *Step* 1.1: *Compute the worst*-*case* *LSP*

6:   **while** Δ ≥ *ε*

7:    **for** $s_{P}\in S_{P}\setminus {\bar{S}}_{P}⋃B_{0}$ **do**

8:     *Min*_*P*_ ← *V*_*π*_(•, *α*)

9:     $V_{\pi }\left(s_{P},\alpha \right)=\min_{P_{P}^{\pi (s_{P})}\in \left[\underline{p},\bar{p}\right]}{\left(P_{P}^{\pi (s_{P})}\right)}^{T}V_{\pi }\left(•,\alpha \right)$

10:     Δ = min(∥*V*_*π*_(•, *α*) − *Min*_*P*_∥, Δ)

11:     **if** Δ ≤ *ε* **then**

12:      $V_{\pi }^{*}\left(s_{P},\alpha \right)=V_{\pi }\left(s_{P},\alpha \right)$

13:     **end if**

14:    **end for**

15:   **end while**

 ▷ *Step* 1.2: *Update the robustness*

16:   *α* ← *α* + 1/*N*

17:   Δ ← ∞

18: **end while**

19: **return** α* ← *α* − 1/*N*

Having formulated the problem and designed the solution algorithms, we will illustrate our methods in the next section.

## 5 Empirical Evaluation

### 5.1 Construction

**Fig 5:**
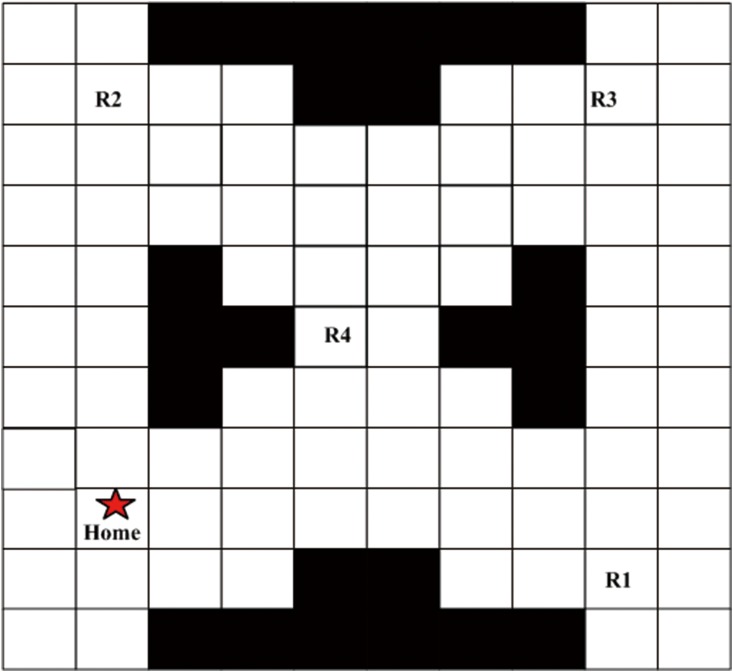
The mission space with atomic propositions.

We demonstrate our algorithms on an example of UAV search missions, in which the task for the UAV is to sequentially visit several regions of interest to collect information while always remaining safe. Once the UAV has visited the regions, it should return to the starting point. Simulation experiments are performed in a warehouse, as shown in Fig 5. The LTL formula for this task is *ϕ* = *G*¬unsafe∧*F*((R1∨R2)∧*XF*(R3∧*XF*(R4∧*XF*home))).

**Fig 6:**
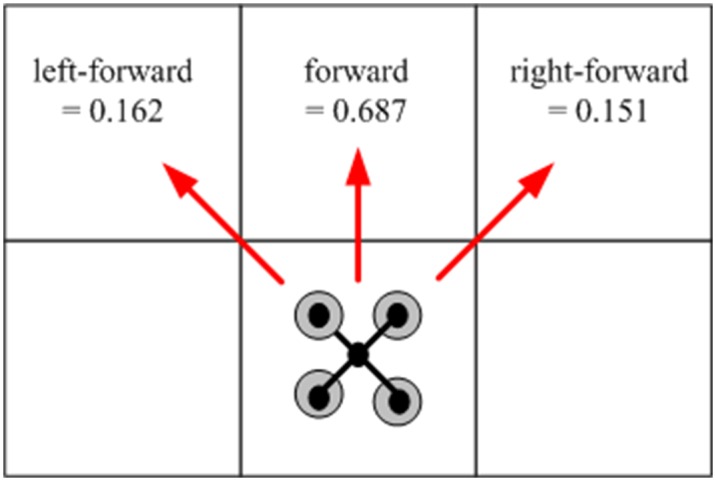
The transition probability for action ‘up’.

Without any loss of generality, the occupancy grid map is utilized to discretize the workspace, which consists of 10 × 11 cells. Each cell is a square region [0, 1] × [0, 1]. Considering a UAV moving in the discrete grid map consisting of 10 × 11 cells, we create a finite MDP abstraction of the UAV system. The UAV is located at one of the cells of the grid map each time, so each cell represents a state of the MDP. The actions enabled at each state are {up, down, left, right}, each of which can direct the UAV to move to one of its three forward adjacent regions. The atomic propositions are {home, R1, R2, R3, R4, unsafe}, each of which labels the states of the MDP with an atomic proposition, as shown in Fig 5. The first four propositions are labeled on the corresponding cells of the grid map, and the unsafe regions are represented by the black blocks in the cells. Due to the symmetry of the region and the UAV, we only need to calculate the estimated transition probability for one region and can apply it to other regions. Through Monte Carlo simulation [27], the transition probabilities for taking action ‘up’ are obtained (*left-forward*, *forward*, *right-forward*) = (0.162, 0.687, 0.151), as shown in Fig 6. The transition probabilities for taking other actions are defined in a similar manner. Note that the estimated transition probabilities are not exact as a result of statistical errors. And some undesired state transitions may be caused by control errors or environmental disturbance.

Computations are performed on a 2.6GHz Intel Core i7 processor with 8 GB memory. All the experiments are implemented in the MATLAB. The LTL formula *ϕ* is transformed into a DRA with 7 states through the ‘ltl2dstar’ software [39], so we can obtain the product IMDP with 770 states by constructing the Cartesian product of the DRA and the IMDP. It takes 0.31s to generate AMECs for the product IMDP.

### 5.2 Implementation and Results

In the following, we will demonstrate our robust satisficing decision-making method in two parts. In the first part, our method is used to generate a robust satisficing policy for the given DLSP, and the effectiveness of this policy is verified by determining whether the DLSP is met. Besides, the trade-off property between robustness and the DLSP is analyzed. In the second and third parts, our method is compared with the min-max robust decision-making method and the robust decision making [37] to illustrate the advantages of the robust satisficing policy in practice.

#### 5.2.1 Generation and Properties of the Robust Satisficing Policy

In this subsection, we will verify the effectiveness of the proposed algorithm. To begin with, the simulation parameters are set as follows. For the DLSP, in some applications it might derive from established standards or rules within an organization. In other cases, the DLSP might derive from a combination of the decision maker’s values and understanding of what is possible. In this paper, we take the second approach, and the DLSP *p*_*c*_ is initially set to be 0.9. And the uncertainty division *N* is set to be 100 such that the accuracy of the robustness is 0.01, which is small enough for the transition probability.

**Fig 7:**
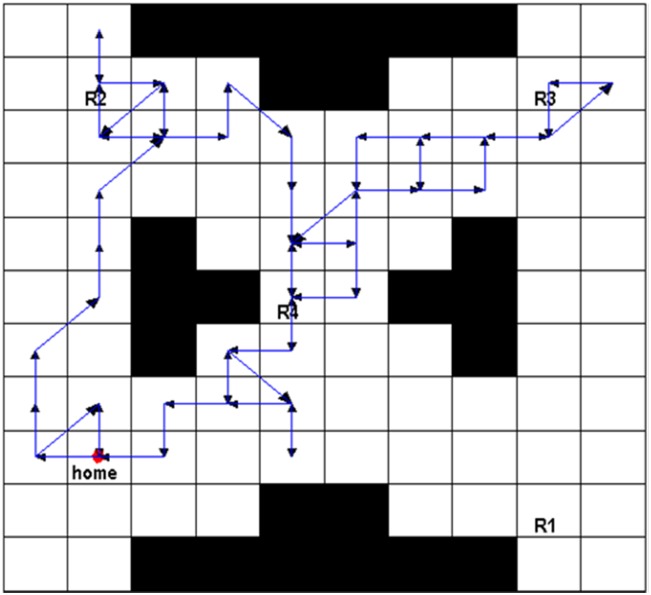
The original trajectory of the robust satisficing policy through forward simulation.

**Fig 8:**
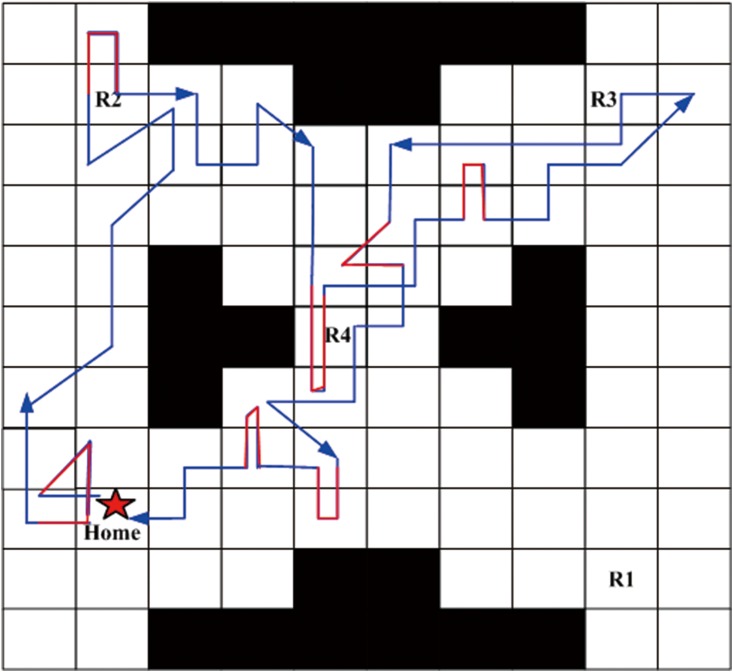
The trajectory of the robust satisficing policy removing overlaps.

Next, a robust satisficing policy is generated by implementing Algorithm 1 in the UAV search mission environment. Aiming at an intuitively expressive form of the resulting policy, we carry out the forward simulation to produce a trajectory of the policy. The UAV starts from the initial state, and determines the action to be taken at the current state according to the generated policy. Then, the transition to the next state is conducted by the worst-case transition probability which can be calculated by the linear programming function (linprog) in the MATLAB. The above procedures are repeated until it reaches a terminal state (AMEC). After the Algorithm 1 is run, the robust satisficing policy π* is generated in 452.8s with a robustness of 0.40. Through the forward simulation, a trajectory of the UAV as shown in Fig 7 can be generated. The star sign represents the start point, the trajectory is drawn with the blue line, and the arrows represent the direction of motion. There are several overlaps of the path segments as shown in Fig 7, which may cause a little fuzziness, so another figure is drawn to remove the overlaps, as shown in Fig 8. The red path segments represent the influence of uncertainty which makes the motions of UAV deviate from the desired direction of action, while our robust satisficing policy can drive the UAV back.

**Fig 9:**
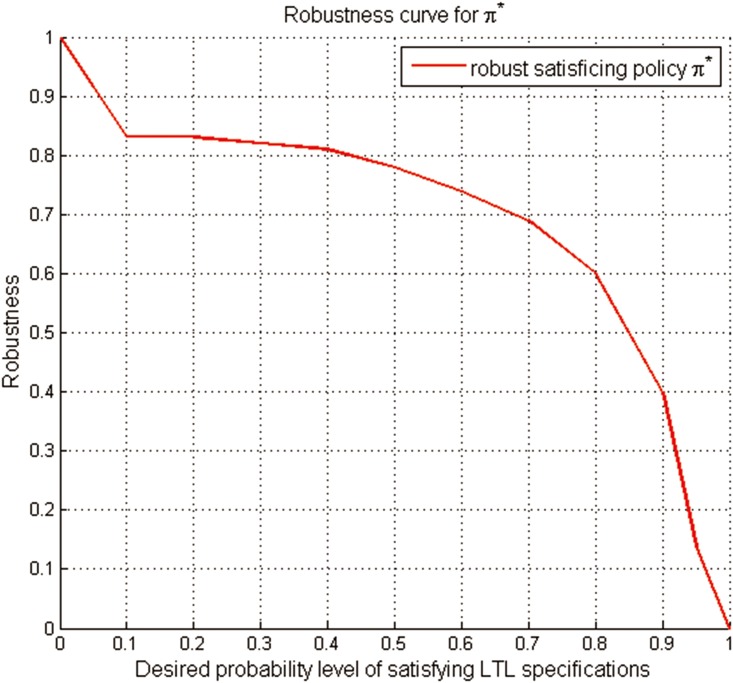
The robustness curve for the robust satisficing policy π*.

**Fig 10:**
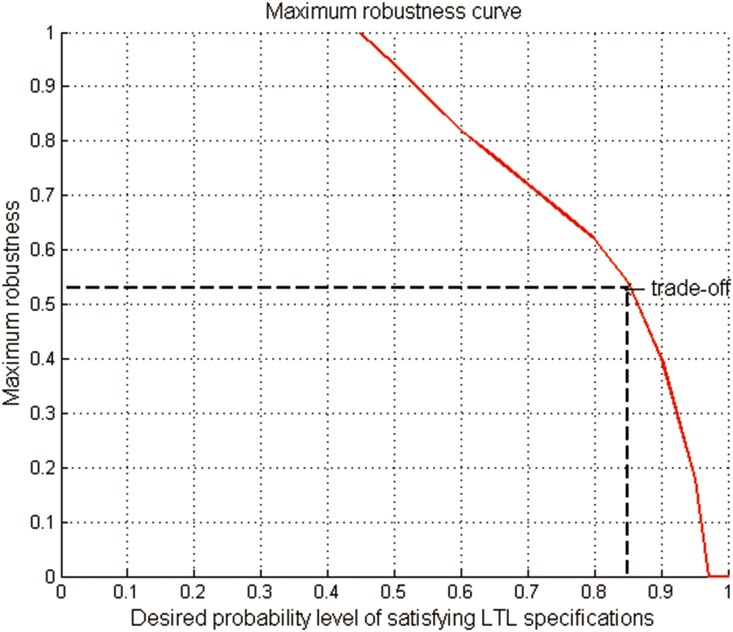
The maximum robustness curve.

In order to describe the trade-off relationship between the DLSP and the robustness, we first consider the effect of different DLSPs on the robustness of the generated robust satisficing policy π*. The robustness of the generated policy π* for all the DLSPs from 0.0 to 1.0 is calculated by implementing Algorithm 2. And a robustness curve of π*, representing its varying robustness corresponding to different DLSPs, is shown in Fig 9. It can be seen that the robustness of π* will decrease as the DLSP increases. Further, we demonstrate the effect of DLSP on the maximum robustness. Considering different DLSPs, we run Algorithm 1 to generate the robust satisficing policy and the corresponding maximum robustness. Fig 10 shows the maximum robustness curve, which represents the relationship between the DLSP and the maximum robustness. And the robustness of π* and the maximum robustness for different DLSPs are shown in Table 1.

**Table 1:** Robustness for different desired probability levels of satisfying LTL specifications.

**DLSP** *p*_*c*_	**0.00**	**0.10**	**0.20**	**0.30**	**0.40**	**0.50**
robustness of π*	1.00	0.83	0.83	0.82	0.81	0.78
maximum robustness	1.00	1.00	1.00	1.00	1.00	0.94
**DLSP** *p*_*c*_	**0.60**	**0.70**	**0.80**	**0.90**	**0.95**	**1.00**
robustness of π*	0.74	0.69	0.60	0.40	0.14	0.00
maximum robustness	0.82	0.72	0.62	0.40	0.18	0.00

From Fig 10, it can be seen that the slope of the DLSP vs. maximum robustness is always negative. This negative slope represents the trade-off in Theorem 3: as the DLSP increases, the robustness to uncertainty naturally decreases. Note that if the DLSP decreases from 1 to 0.8, the robustness will increase sharply from 0 to around 0.6. Yet, if the DLSP continuously decreases, the increase of the robustness will not be remarkable. Thus we recommend a more appropriate DLSP (around 0.85) to the UAV operator instead of 0.9, which can be explained by the principle that a little compromise on performance can lead to a relatively obvious increase in robustness.

Second, we demonstrate that the robust satisficing policy at the maximum uncertainty level (i.e., robustness), will always guarantee the DLSP satisfied. Through Monte Carlo simulation, the true LSP of a resulting policy is computed as the mission success rate over 1000 independent forward simulation runs. Four robust satisficing policies (*π*_1_, …, *π*_4_) with robustness (*α*_1_, … *α*_4_) for four different DLSPs (0.9, 0.8, 0.7, 0.6) are generated, respectively. For each of these policies, the true LSP is calculated through Monte Carlo simulation and will be compared with its corresponding DLSP. The results are shown in Table 2. The first row shows the DLSP *p*_*c*_; in the second row are the robust satisficing policies, with the robustness in the third row; and in the last row are the true LSPs obtained through Monte Carlo simulations. It can be observed that for each of these policies, the true LSP is higher than its corresponding DLSP. So the robust satisficing policies generated by Algorithm 2 can guarantee the DLSP satisfied.

**Table 2:** Desired probability level vs. true probability of satisfying the LTL specifications.

DLSP *p*_*c*_	0.90	0.80	0.70	0.60
robust satisficing policy	*π*_1_	*π*_2_	*π*_3_	*π*_4_
robustness	0.40	0.62	0.72	0.82
true LSP	0.955	0.947	0.788	0.613

#### 5.2.2 Comparison with the Min-Max Robust Decision-Making Method

**Fig 11:**
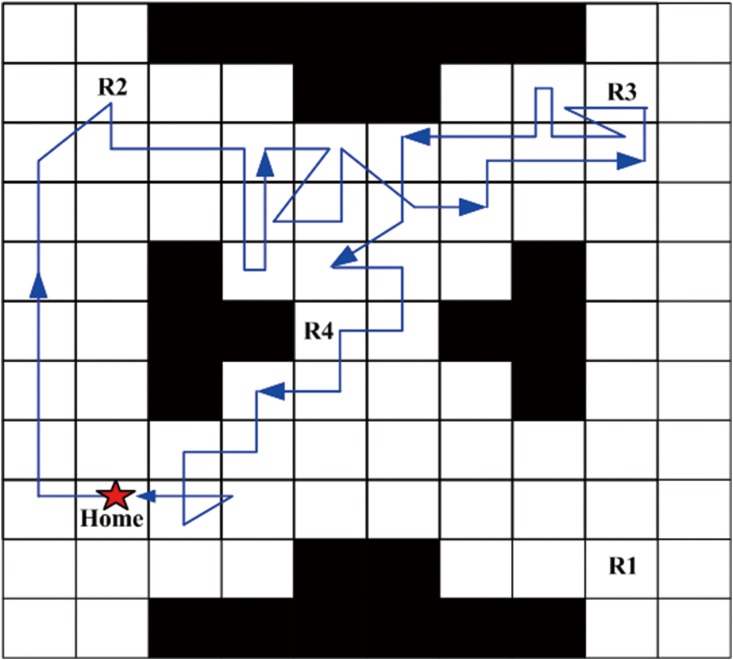
The trajectory of the min-max robust policy *π*_*m*_ through forward simulations.

**Fig 12:**
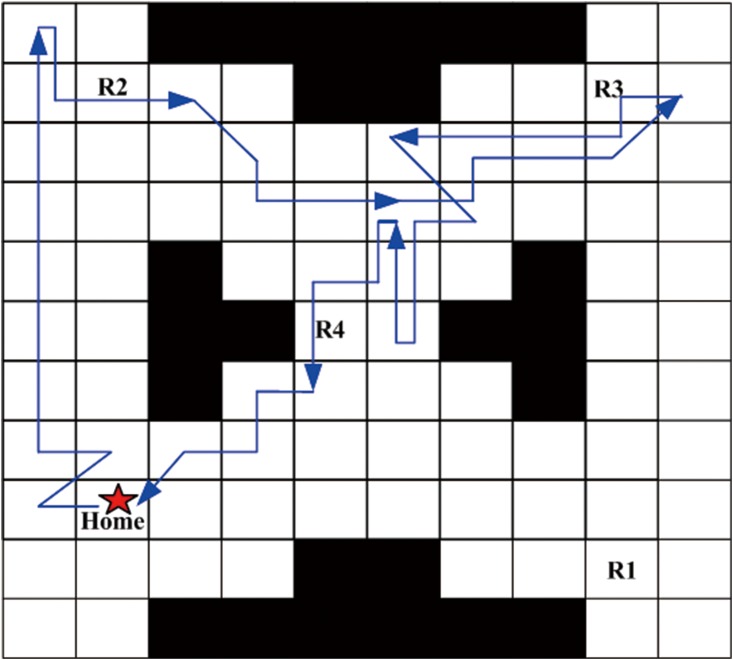
The trajectory of the robust satisficing policy *π*_*s*_ through forward simulations.

**Fig 13:**
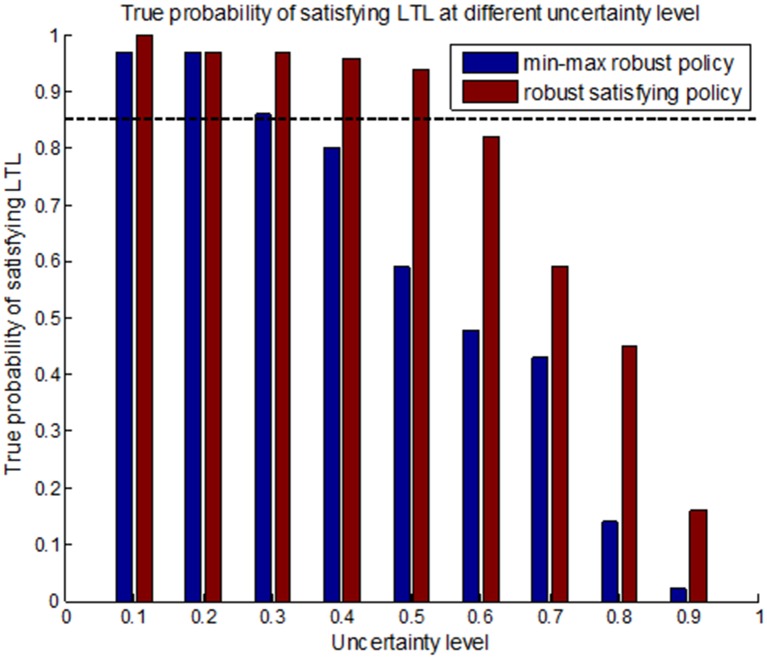
The histograms representing the true LSPs for the min-max robust policy *π*_*m*_ and the robust satisficing policy *π*_*s*_ at different uncertainty levels.

**Fig 14:**
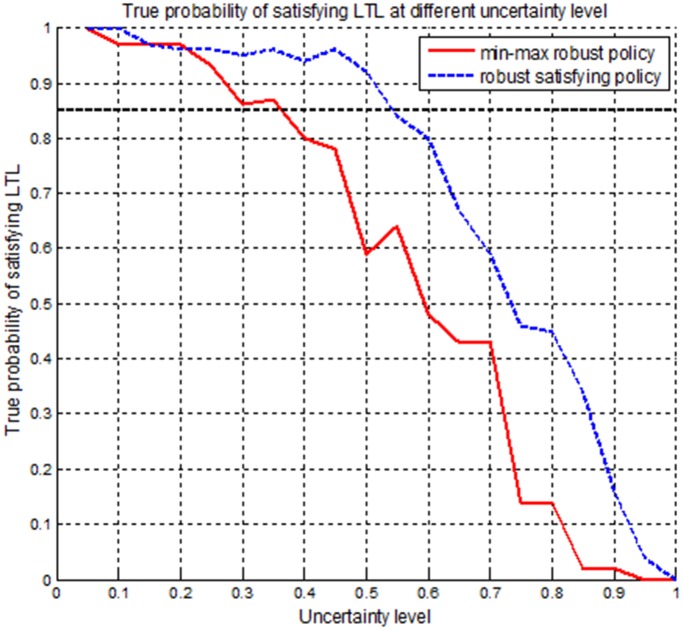
The curves representing the true LSPs for the min-max robust policy *π*_*m*_ and the robust satisficing policy *π*_*s*_ at different uncertainty levels.

In this subsection, we will compare our robust satisficing decision-making method with the min-max robust decision-making method [27] in real UAV applications. It is assumed that the desired mission success rate (i.e., DLSP) is *p*_*c*_ = 0.85 for the UAV, which is the trade-off performance obtained from the robustness curve. The environment and mission specifications are set identically in Subsection 5.1. The estimated uncertainty level of transition probability is initially assumed to be 0.2. By using the min-max robust decision-making method, a robust policy *π*_*m*_ can be generated at the estimated uncertainty level, and the trajectory generated by forward simulations is shown in Fig 11. Then our robust satisficing decision-making method is used to generate a robust satisficing policy *π*_*s*_ for the DLSP *p*_*c*_ = 0.85. The resulting policy *π*_*s*_ has a robustness of 0.54, and the trajectory is shown in Fig 12. The true LSPs for these two policies are calculated through Monte Carlo simulations, and the values are 0.97 and 0.96 for *π*_*m*_ and *π*_*s*_, respectively. It can be seen that if the true uncertainty level is 0.2, both *π*_*m*_ and *π*_*s*_ satisfy the DLSP. However, in real applications, the estimated uncertainty level may be inaccurate as a result of environment disturbance. In order to determine the failure boundaries for *π*_*m*_ and *π*_*s*_, their true LSPs are evaluated at different uncertainty levels from 0.1 to 1, and the results are shown as the histograms and curves in Figs 13 and 14, as well as the data in Table 3.

**Table 3:** The true LSPs for the min-max robust policy *π*_*m*_ and the robust satisficing policy *π*_*s*_ at different uncertainty levels.

**uncertainty level**	**0.00**	**0.10**	**0.20**	**0.30**	**0.40**	**0.50**
LSP for *π*_*m*_	1	0.97	0.97	0.86	0.80	0.59
LSP for *π*_*s*_	1	1	0.96	0.95	0.94	0.92
**uncertainty level**	**0.60**	**0.70**	**0.80**	**0.90**	**0.95**	**1.00**
LSP for *π*_*m*_	0.48	0.43	0.14	0.02	0.00	0
LSP for *π*_*s*_	0.80	0.59	0.45	0.16	0.04	0

From Figs 13 and 14 and Table 3, it can be seen that if the uncertainty level exceeds 0.37, the min-max robust policy *π*_*m*_ will lead to a value smaller than 0.85 such that it cannot guarantee the DLSP. However, for the robust satisficing policy *π*_*s*_, the DLSP will be met until the uncertainty level extends to 0.54. And from the curves, it can be observed that the true LSP of the robust satisficing policy is higher than that of the min-max robust policy at all the uncertainty levels except from 0.15 to 0.21. Therefore, it can be concluded that our robust satisficing policy can tolerate higher uncertainty than the min-max robust policy, and is much more effective in real applications to guarantee a desired performance level.

#### 5.2.3 Comparison with Robust Decision Making

Considering the robustness optimization problem under severe uncertainty, we compare the proposed method with the robust decision making (RDM) [37, 40], which also provides a structured approach to making robust decisions under severe uncertainty. The RDM employs two decision criteria, i.e., definitions of robustness, adopted in the risk analysis literature [40, 41]. The first defines a robust policy as one that trades some optimal performance for less sensitivity to uncertainty, namely the limited degree of confidence (LDC) criterion. And the second defines a robust policy as one that performs relatively well compared with the alternatives over a wide range of futures, namely the wide range of futures (WRF).

**Fig 15:**
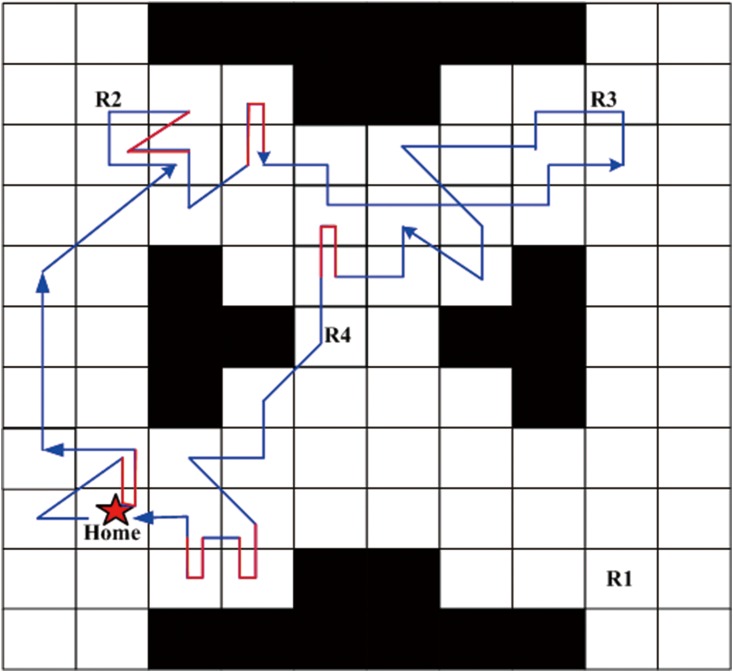
The trajectory of the RDM robust policy *π*_*r*1_ through forward simulations.

**Fig 16:**
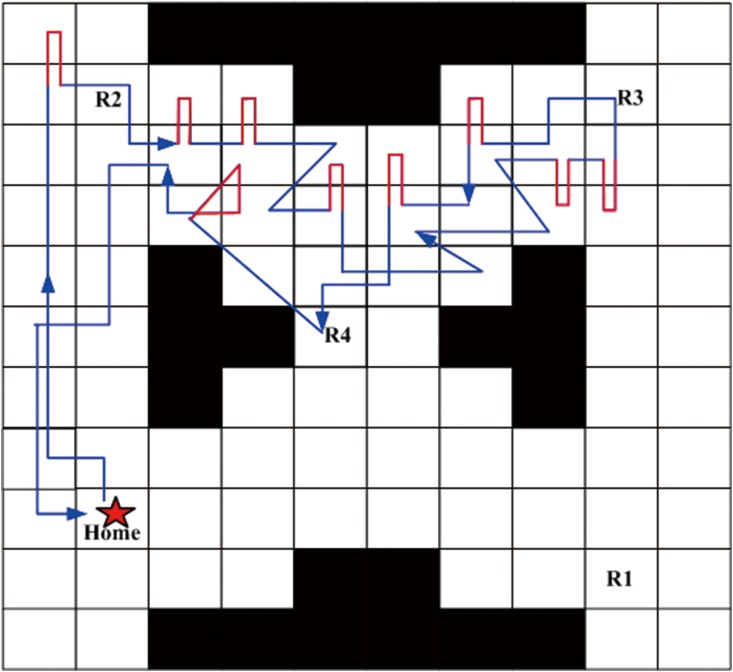
The trajectory of the RDM robust policy *π*_*r*2_ through forward simulations.

**Fig 17:**
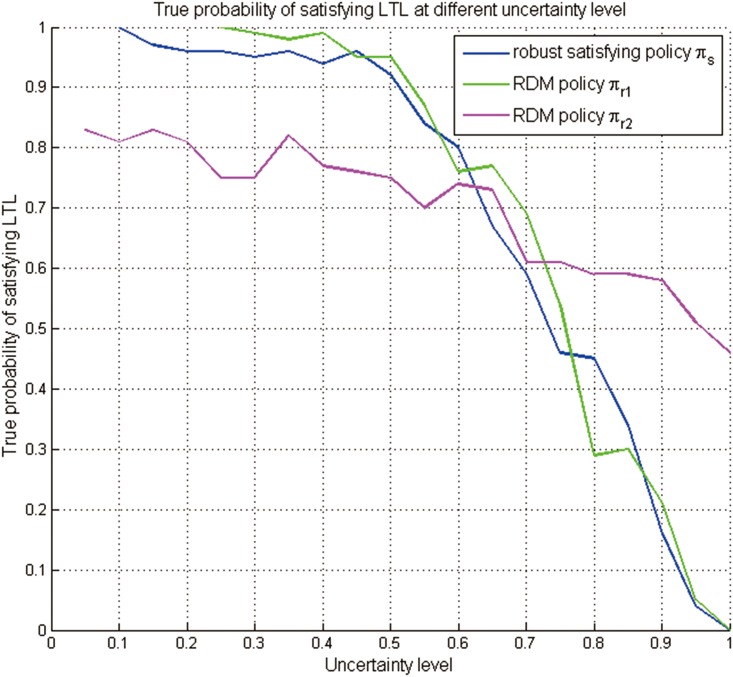
The curves representing the true LSPs for the robust satisficing policy *π*_*s*_ and the RDM robust policies *π*_*r*1_ and *π*_*r*2_ at different uncertainty levels.

With respect to the above two robustness criteria, two RDM robust policies *π*_*r*1_ and *π*_*r*2_ are generated by the robust decision making [41], and the trajectories are shown in Figs 15 and 16. In order to compare the robust satisficing policy *π*_*s*_ (generated by our method for the DLSP *p*_*c*_ = 0.85 in the previous subsection) with the RDM robust policies *π*_*r*1_ and *π*_*r*2_, their true LSPs are evaluated at different uncertainty levels from 0.1 to 1, and the results are shown as the curves in Fig 17 and the data in Table 4.

**Table 4:** The true LSPs for the robust satisficing policy *π*_*s*_ and the RDM robust policies *π*_*r*1_ and *π*_*r*2_ at different uncertainty levels.

**uncertainty level**	**0.00**	**0.10**	**0.20**	**0.30**	**0.40**	**0.50**
LSP for *π*_*s*_	1	1	0.96	0.95	0.94	0.92
LSP for *π*_*r*1_	1	1	1	0.99	0.99	0.95
LSP for *π*_*r*2_	0.85	0.81	0.81	0.75	0.77	0.75
**uncertainty level**	**0.60**	**0.70**	**0.80**	**0.90**	**0.95**	**1.00**
LSP for *π*_*s*_	0.80	0.59	0.45	0.16	0.04	0
LSP for *π*_*r*1_	0.76	0.69	0.29	0.21	0.05	0
LSP for *π*_*r*2_	0.70	0.61	0.59	0.58	0.51	0.46

From Fig 17 and Table 4, it can be seen that the RDM robust policy *π*_*r*1_ performs almost the same as the robust satisficing policy *π*_*s*_, since it defines robustness as trading some optimal performance for less sensitivity to uncertainty, which is similar to the info-gap decision theory. The RDM robust policy *π*_*r*2_ has the lowest LSP when the range of uncertainty level is from 0 to 0.6, and when the uncertainty level increases above 0.7, it will have the highest LSP. The RDM robust policy *π*_*r*2_ performs relatively well over all uncertainty levels, for it defines robustness as performing relatively well over a wide range of plausible futures. Our method begins with the estimated value for each uncertain system input and then sequentially chooses values increasingly farther away from the expected inputs, while the RDM samples all the possible uncertain parameters to identify the conditions of system failure. In this problem, it is not difficult to obtain an estimated value of the uncertain transition probability via Monte Carlo simulations. Thus, with the estimated value and a predefined performance level, our method will be more applicable. Given the diversity of definitions of robustness, and the differing judgments called for in implementing alternative robust decision methods, it is perhaps surprising they often reach similar results. Therefore, our method provides an alternative way to make robust decisions to handle the severe uncertainty, like the RDM method.

## 6 Conclusion and Future Work

In this paper, the robust satisficing decision-making problem for complex missions was formulated as the robust synthesizing control problem for an uncertain MDP with the LTL specifications. Based on the info-gap decision theory, we proposed a robust satisficing decision-making method to maximize the robustness to uncertain transition probabilities of the MDP, while guaranteeing the desired probability level of satisfying the LTL specifications. In order to compute the probability of satisfying the LTL specifications, we constructed a product IMDP, which combined the IMDP model representing the uncertain MDP with the DRA converted from the LTL specifications. And a robust satisficing policy generation algorithm based on robust dynamic programming was proposed to solve the robust satisficing decision-making problem, as well as a robustness evaluation algorithm. The algorithm was demonstrated by the simulation results.

The proposed method mainly focuses on the decision-making problem with the following characteristics: the uncertain system model, such as the uncertainty of actuation consequence, which can be modeled as a MDP; deep uncertainty, in which the decision makers does not know or agree on the probability distribution of the key parameters of the model, which can be addressed by the info-gap decision theory; complex missions with rigorous temporal constraints, which can be described by LTL. Potential applications for our method include persistent surveillance task [42], cargo transportation in rough terrain [12], navigation in dangerous environments, and autonomous operation of robots in chaotic work and home environments.

In the future, our method can be extended to many fields of applications. In the field of autonomous robots, we will extend our method to address the multi-robot task cooperation problem with multiple tasks, by introducing the timed automaton and the coordination mechanism for multiple tasks. Besides, the robust satisficing decision making method can be creatively applied to model the severe uncertainty in the economic and climate fields, which can help to generate some robust satisficing economic or climate policies to avoid significant failures.

## Supporting Information

S1 Appendix Inner minimization problem. For solving the inner minimization problem, the dual linear programming is used.
(PDF)

S1 Data MDP data. (XLSX)

S2 Data DRA data. The LTL formula in the experiment is transformed into a DRA.
(XLSX)

S3 Data Generated policies data. This data set includes the robust satisficing policies *π*_1_, *π*_2_, *π*_3_, *π*_4_, and *π*_*s*_, the min-max robust policy *π*_*m*_, and the RDM robust policies *π*_*r*1_ and *π*_*r*2_.
(XLSX)
