# Supplementary material for: Robust Satisficing Decision Making for Unmanned Aerial Vehicle Complex Missions under Severe Uncertainty
Source: PLoS One. 2016 Nov 11;11(11):e0166448. doi: 10.1371/journal.pone.0166448 (PMC5105955; doi:10.1371/journal.pone.0166448)
Supplement: S1 Appendix — For solving the inner minimization problem, the dual linear programming is used. (PDF) [file pone.0166448.s001.pdf]

S1 Appendix.

Inner minimization problem. For solving the inner minimization problem, the dual linear programming is used.

We simplify the inner minimization problem as:

$$\sigma^* = \min_p v^T p : \ p^T \mathbf{1} = 1, \ \underline{p} \leq p \leq \bar{p} \tag{1}$$

where v represents the value function $V_\pi(\bullet, \alpha)$ during each iteration, and p represents a row of transition matrix P^a.

The optimization Eq (1) is a linear, feasible programming. The optimal value can be computed through dual linear programming. Equation (1) can be written as

$$\sigma^* = \min_p v^T p.s.t.p^T \mathbf{1} = 1, \ -p \geq -\bar{p}, \ p \geq \underline{p} \tag{2}$$

Due to the standard principle of dual linear programming, we can obtain the dual function

$$D\sigma^* = \max_{\mu, \lambda_1, \lambda_2} \mu - \bar{p}^T \lambda_1 + \underline{p}^T \lambda_2.s.t.\mu \mathbf{1} - \lambda_1 + \lambda_2 \leq v, \lambda_1 \geq 0, \lambda_2 \geq 0 \tag{3}$$

where $D\sigma^* : \mathbb{R} \times \mathbb{R}^n \times \mathbb{R}^n \to \mathbb{R}$. According to properties of dual transformation, the optimal value for the dual problem is also the optimal value for the original one. So we will describe the method in details to compute the optimal value of the dual linear problem.

Equation (3) is transformed into the following form

$$D\sigma^* = \max_{\mu, \lambda_1, \lambda_2} \mu - \bar{p}^T (\lambda_1 - \lambda_2) - (\bar{p} - \underline{p})^T \lambda_2.s.t.\lambda_1 - \lambda_2 \geq \mu \mathbf{1} - v, \lambda_1 \geq 0, \lambda_2 \geq 0 \tag{4}$$

In order to maximize $D\sigma^*$, $\lambda_1 - \lambda_2$ should have a minimal value of $\mu \mathbf{1} - v$. Inserting $\lambda_1 - \lambda_2 = \mu \mathbf{1} - v$ in Eq (4), we have

$$D\sigma^* = \max_{\mu,\lambda_1\geq 0,\lambda_2\geq 0} \mu - \bar{p}^T(\mu\mathbf{1} - v) - (\bar{p} - \underline{p})^T \lambda_2 \tag{5}$$

Then, we insert $\lambda_2 = \lambda_1 - \mu\mathbf{1} + v \geq 0$ in Eq (5), and have

$$D\sigma^* = \max_{\mu,\lambda_1} \mu - \bar{p}^T(\mu\mathbf{1} - v) - (\bar{p} - \underline{p})^T(\lambda_1 - \mu\mathbf{1} + v).s.t.\lambda_1 \geq 0, \lambda_1 \geq \mu\mathbf{1} - v \tag{6}$$

It can be seen that λ_1 should be as small as possible while satisfying the inequality constraints.

- If $\mu\mathbf{1} - v \leq 0$, the optimal λ_1 is 0, we have
  $D\sigma^* = \max_\mu \mu(1 - \bar{p}^T\mathbf{1}) + \bar{p}^T v + (\bar{p} - \underline{p})^T(\mu\mathbf{1} - v)$.
- If $\mu\mathbf{1} - v \geq 0$, the optimal λ_1 is $\mu\mathbf{1} - v$, we have $D\sigma^* = \max_\mu \mu(1 - \bar{p}^T\mathbf{1}) + \bar{p}^T v$.

Combining the above two cases, we obtain a simplified expression of the maximal value

$$D\sigma^* = \max_{\mu} \mu(1 - \bar{p}^T\mathbf{1}) + \bar{p}^T v + (\bar{p} - \underline{p})^T \min\{(\mu\mathbf{1} - v), 0\} \tag{7}$$

where $\min\{x, y\}$ represents the smaller one between x and y. The dual optimization problem is a convex piecewise linear function with break points $v(i)$, $i = 1, \ldots, n$. It can be seen that the maximal value can be attained at one of the break points.
